# Involvement of Siglec-15 in regulating RAP1/RAC signaling in cytoskeletal remodeling in osteoclasts mediated by macrophage colony-stimulating factor

**DOI:** 10.1038/s41413-024-00340-w

**Published:** 2024-06-07

**Authors:** Hideyuki Kobayashi, M. Alaa Terkawi, Masahiro Ota, Tomoka Hasegawa, Tomomaya Yamamoto, Tomohiro Shimizu, Dai Sato, Ryo Fujita, Toshifumi Murakami, Norio Amizuka, Norimasa Iwasaki, Masahiko Takahata

**Affiliations:** 1 Department of Orthopaedic Surgery, Faculty of Medicine and Graduate School of Medicine, Hokkaido University, Sapporo, 060-8638 Japan; 2 Department of Developmental Biology of Hard Tissue, Graduate School of Dental Medicine, Hokkaido University, Sapporo, Japan; 3 Department of Orthopaedic Surgery, Dokkyo Medical University, Mibu Shimotsuga, 321-0293 Japan

**Keywords:** Bone, Osteopetrosis

## Abstract

DNAX-associated protein 12 kD size (DAP12) is a dominant immunoreceptor tyrosine-based activation motif (ITAM)-signaling adaptor that activates costimulatory signals essential for osteoclastogenesis. Although several DAP12-associated receptors (DARs) have been identified in osteoclasts, including triggering receptor expressed on myeloid cells 2 (TREM-2), C-type lectin member 5 A (CLEC5A), and sialic acid-binding Ig-like lectin (Siglec)-15, their precise role in the development of osteoclasts and bone remodeling remain poorly understood. In this study, mice deficient in *Trem-2*, *Clec5a*, *Siglec-15* were generated. In addition, mice double deficient in these DAR genes and FcεRI gamma chain (FcR)γ, an alternative ITAM adaptor to DAP12, were generated. Bone mass analysis was conducted on all mice. Notably, *Siglec-15* deficient mice and *Siglec-15/FcRγ* double deficient mice exhibited mild and severe osteopetrosis respectively. In contrast, other DAR deficient mice showed normal bone phenotype. Likewise, osteoclasts from *Siglec-*15 deficient mice failed to form an actin ring, suggesting that Siglec-15 promotes bone resorption principally by modulating the cytoskeletal organization of osteoclasts. Furthermore, biochemical analysis revealed that Sigelc-15 activates macrophage colony-stimulating factor (M-CSF)-induced Ras-associated protein-1 (RAP1)/Ras-related C3 botulinum toxin substrate 1 (Rac1) pathway through formation of a complex with p130CAS and CrkII, leading to cytoskeletal remodeling of osteoclasts. Our data provide genetic and biochemical evidence that Siglec-15 facilitates M-CSF-induced cytoskeletal remodeling of the osteoclasts.

## Introduction

Osteoclasts are bone-resorbing cells that play a pivotal role in skeletal development since they are the major effector cells involing in bone remodeling, both physiological and pathological conditions. Growth factors, namely macrophage colony-stimulating factor (M-CSF) and receptor activator of nuclear factor κB ligand (RANKL), essentially mediate differentiation of hematopoietic precursors of the monocyte/macrophage lineage into mature multinucleated osteoclasts (osteoclastogenesis).^1^ This requires, in parallel, costimulatory signaling through immunoreceptor tyrosine-based activation motif (ITAM)-signaling adaptors, DNAX-associated protein 12 kD size (DAP12), and FcεRI gamma chain (FcRγ).^2,3^ Importantly, cells from DAP12 and FcRγ double deficient mice display impaired osteoclastogenesis, emphasising the importance of this costimulatory signaling in cell differentiation and maturation.

The lack of adequate extracellular domains in DAP12 and FcRγ, which are necessary for sensing signals outside the cells, suggests that ITAM signaling requires activation of certain immunoreceptors associated with either DAP12 or FcRγ.^4,5^ Recent evidence using transgenic mice suggests that DAP12-associated receptors (DARs) play a dominant role in osteoclastogenesis.^2,6^ Sialic acid-binding Ig-like lectin (Siglec)-15, triggering receptor expressed in myeloid cells (TREM-2) and myeloid DAP12-associated lectin (MDL-1) are to date known as potential regulators of osteoclastogenesis.^7–12^ It is also evident that FcRγ partially compensates for the absence of DAP12 and mediates the activation of ITAM signaling in DAR-deficient mice that rescues bone phenotype in physiological bone remodeling.^2,3^ Siglec-15 seems to be the most important DAR as *Siglec-15* deficient mice have a mild osteopetrotic phenotype which has not been demonstrated in mice lacking other known DARs.^7,13^ Siglec-15 modulates RANKL-induced phosphatidylinositol 3‐kinase/Akt and Erk pathways required for osteoclast differentiation and maturation. Moreover, deletion of *Siglec-15* does not alter transcriptional regulation by nuclear factor of activated T-cells (NFAT)-c1 and nuclear factor (NF)κB, which are key transcriptional regulators downstream of RANKL signaling, or influence differentiation of osteoclasts; however, mononuclear osteoclasts of *Siglec-15* defeicnt mice are not able to form actin rings.^7^ These findings together underline the primary role of the Siglec-15/DAP12 pathway as a mediator of cytoskeletal organization of osteoclasts, but not as a facilitator of osteoclast recruitment/maturation. However, the precise mechanism by which Siglec-15 regulates cytoskeletal organization is poorly understood. Given the fact that cytoskeletal organization is mediated by M-CSF signaling via activation of the αvβ3 integrin-DAP12-Syk axis,^14,15^ there is possibility that Siglec-15 is involved in this signaling cascade. Therefore, a better understanding of the impaired cytoskeletal organization in pathological bone resorption requires further investigation of the precise role of Siglec-15 in pathological bone resorption and its involvement in cytoskeletal organization.

Given this background, the current study was designed to determine whether Siglec-15 is a critical DAR of osteoclasts in the skeletal system using DARs and FcRγ double-deficient mice, and investigate the molecular mechanism by which the Siglec-15/DAP12 axis regulates cytoskeletal organization of osteoclasts mediated by M-CSF-signaling.

## Results

### Siglec-15 is a critical DAR in the maintenance of bone mass via regulating maturation of functional osteoclasts in secondary spongiosa

**Fig. 1:**
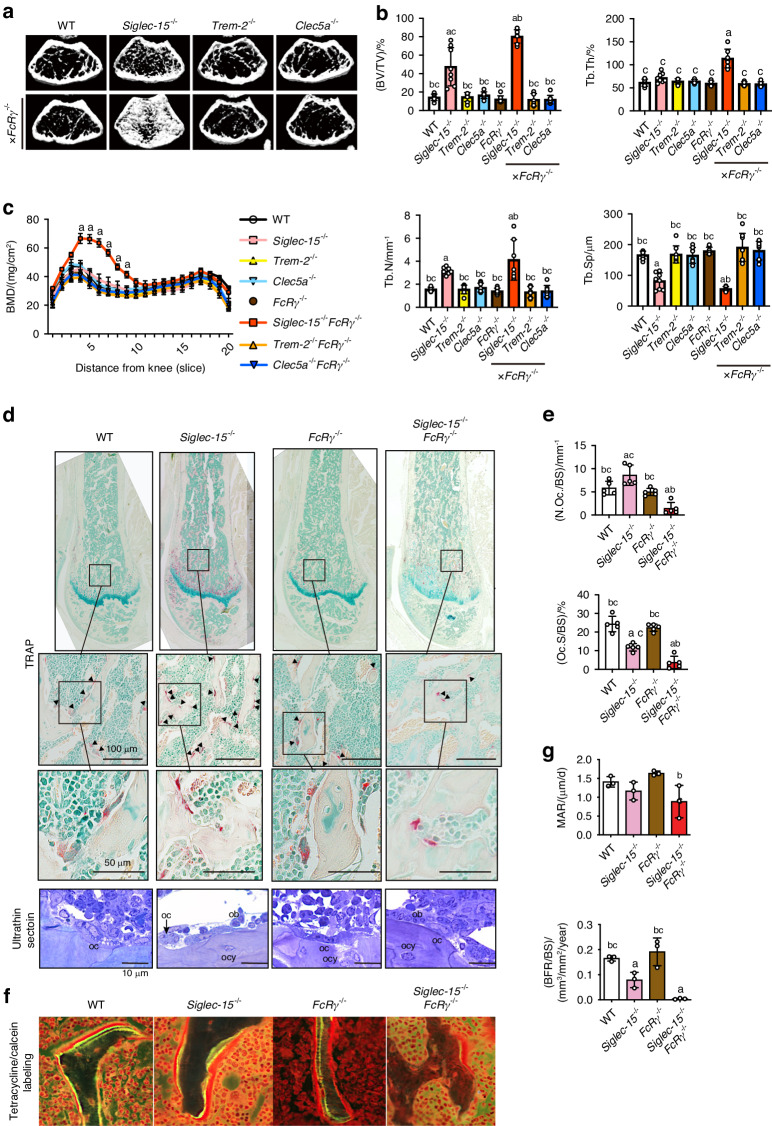
*Siglec-15* deficient mice exhibit osteopetrosis. *Siglec-15*^*−/*^^−^ mice exhibit mild osteopetrosis and *Siglec-15*^−/−^*FcRγ*^−/−^ mice display severe osteopetrosis, whereas other DAR-deficient mice (*FcRγ*^−/−^, *Trem-2*^−/−^*FcRγ*^−/−^, and *Clec5a*^−/−^*FcRγ*^−/−^) exhibit normal phenotype. **a** Representative axial cross-sectional images of micro-computed tomography (μCT) of the distal metaphysis of a 14-week-old femur. **b** Bone volume and microstructural indices of trabecular bone of the metaphyseal region of the distal femur. BV/TV; bone volume/total volume, Tb.Th; trabecular thickness, Tb. N; trabecular number, Tb. Sp; trabecular separation (*n* = 10 per group). **c** Bone mineral density (BMD) in 20 longitudinal femur divisions by dual-energy X-ray absorptiometry (DXA) (*n* = 3 per group). **d** Micrographs of the distal femur stained with tartrate-resistant acid phosphatase (TRAP) for osteoclasts (upper raw) and the magnified images of the secondary spongiosa of the distal metaphysis (middle raw). Toluidine blue staining (lower panels) shows that *Siglec-15*^−/−^ and *Siglec-15*^−/−^*FcRγ*^−/−^ osteoclasts (oc) do not spread on bone surface. Ob; osteoblast, ocy; osteocytes. **e** Bone histomorphometry analysis of osteoclast number and osteoclast surface/bone surface (N.Oc/BS and Oc.S/BS) at the secondary spongiosa of the distal femur (*n* = 5 per group). **f** Fluorescence micrographs showing reduced tetracycline and calcein labeling surface especially in *Siglec-15*^−/−^*FcRγ*^−/−^ mice. **g** Dynamic bone histomorphometry analysis of the proximal tibia. MAR; mineral apposition rate, BFR; bone formation rate (*n* = 3 per group). Data are shown as the mean ± S.D. Significant differences among the groups were determined by one-way ANOVA, followed by Tukey’s multiple-comparison procedure. ^a^*P* < 0.05 vs WT mice group; ^b^*P* < 0.05 vs *Siglec-15*^−/−^ mice group; ^c^*P* < 0.05 vs *Siglec-15*^−/−^*FcRγ*^−/−^ mice group

To investigate which DAR plays a dominant role in physiological bone remodeling, mice deficient in one of the DAR genes namely *Siglec-15, Trem-2* and *Clec5a*, and double deficient in one of these genes plus *FcRγ* were generated. Bone mass parameters in the distal metaphysis of the femur from the 7 strains of generated deficient mice (*Siglec-15*^−/−^, *Trem-2*^−/−^, *Clec5a*^−/−^, *FcRγ*^−/−^*, Siglec-15*^−/−^*FcRγ*^−/−^*, Trem-2*^−/−^*FcRγ*^−/−^*, Clec5a*^*-/*^ *FcRγ*^−/−^^*-*^), as well as wild type (WT) mice were determined at 14-week-old. Micro-computed tomography (CT) imaging revealed that there was a mild increase in the trabecular bone mass of the distal femur of *Siglec-15*^−/−^ mice, but not in that of the *Trem-2*^−/−^ and *Clec5a*^−/−^ mice, when compared to that of the WT mice (Fig. 1a). Likewise, *Siglec-15*^−/−^*FcRγ*^−/−^ mice exhibited osteopetrosis features that appeared more severe than those in *Siglec-15*^−/−^ mice as evidenced by the trabecular bone volume to total volume (BV/TV), trabecular thickness (Tb.Th), trabecular number (Tb.N), and trabecular separation (Tb.Sp) values (Fig. 1a, b). In line with the micro-CT results, dual-energy X-ray absorptiometry (DXA) of the femur showed an increase in bone mineral density (BMD) in *Siglec-15*^−/−^*FcRγ*^−/−^ compared with that in other groups (Fig.1c). These results suggest that Siglec-15 is a critical DAR in the maintenance of bone mass. To further confirm our findings, histological examinations of the distal femur were performed. Of note, the number of tartrate-resistant acid phosphatase (TRAP)-positive cells was significantly decreased in the secondary spongiosa of *Siglec-15*^−/−^*FcRγ*^−/−^ mice (Fig. 1d, e). *Siglec-15*^−/−^ mice exhibited decreased osteoclasts surface/bone surface (Oc.S/BS) in secondary spongiosa compared to that in WT mice (Fig. 1d, e). These collectively imply that *Siglec-15* deficiency impaired physiological bone resorption activity of osteoclasts. *Trem-2*^−/−^, *Clec5a*^−/−^*, Trem-2*^−/−^*FcRγ*^−/−^, and *Clec5a*^−/−^*FcRγ*^−/−^ mice did not show significant changes in osteoclast number and size compared with that of WT mice (Fig. S1). Given the coupling bone resorption and formation, we next evaluated bone formation by dynamic bone histomorphometry using non-demineralized sections of the proximal tibia. There was no significant difference in mineral apposition rate (MAR) in WT, *Siglec-15*^−/−^, and *Siglec-15*^−/−^*FcRγ*^−/−^ mice, whereas the bone formation rate (BFR) was significantly decreased in *Siglec-15*^−/−^ mice, and more severely decreased in *Siglec-15*^−/−^*FcRγ*^−/−^ mice, indicating low bone turnover coupled with impaired bone resorption in *Siglec-15*^−/−^ and *Siglec-15*^−/−^*FcRγ*^−/−^ mice (Fig. 1f, g).

### Siglec-15 and FcRγ regulate osteoclast development in primary spongiosa and are essentially involved in skeletal growth

**Fig. 2:**
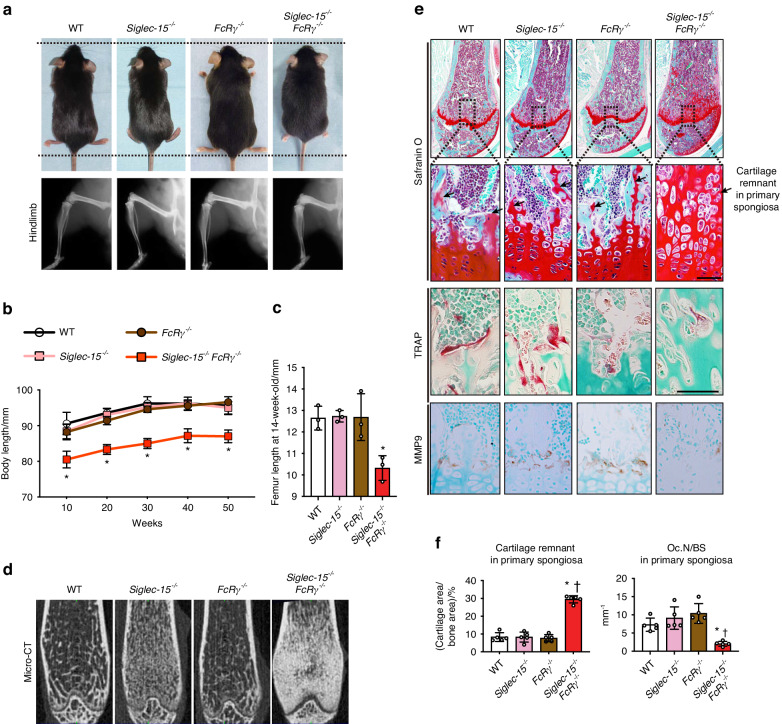
Mice deficient in *Siglec-15* and *FcRγ* display dwarfism and abnormal growth features due to defective replacement of calcified cartilage by bone at the growth plate. **a** Representative images for mice and gross appearance and X-ray of 10-week-old male mice (*n* = 5). **b** Quantification of the body length of the mice. **c** Quantification of the femur length of the mice (*n* = 5). **d** Representative coronal cross-sectional µCT images of distal femora. **e** Micrographs of the chondro-osseous junction and primary spongiosa of distal femur show abundant cartilage remnants (arrows) in metaphyseal region, which is normally resorbed and replaced by bone, in *Siglec-15*^−/−^*FcRγ*^−/−^ mice. There were significantly fewer TRAP-positive cells and matrix metalloproteinase (MMP)-9-positive cells at the cartilage-to-bone transition zone in *Siglec-15*^−/−^*FcRγ*^−/−^ mice than in mice of the other groups. MMP-9-positive cells represent osteoclast-like cells, which can resorb cartilage. **f** Quantitative analysis of cartilage remnants per trabecular bone area and osteoclast number per bone surface (N.Oc/BS) in primary spongiosa of the distal femur (*n* = 5 per group). Data are shown as the mean ± S.D. Significant differences among the groups were determined by one-way ANOVA, followed by Tukey’s multiple-comparison procedure. ^a^*P* < 0.05 vs WT mice group; ^b^*P* < 0.05 vs *Siglec-15*^−/−^ mice group; ^c^*P* < 0.05 vs *Siglec-15*^−/−^*FcRγ*^−/−^ mice group; ^d^*P* < 0.05 vs *FcRγ*^−/−^ mice group

Given the correlation between osteoclast differentiation and skeletal growth,^16,17^ we investigated body length and weight of the deficient mice longitudinally for a period of one year starting at 10 weeks of age. Results revealed that *Siglec-15*^−/−^*FcRγ*^−/−^ mice exhibited dwarfism features evidenced by shorter body length than WT, *Siglec-15*^−/−^ and *FcRγ*^−/−^ mice (Fig. 2a, b). It is worth mentioning that *Siglec-15*^−/−^*FcRγ*^−/−^ mice exhibited no defects in tooth eruption (data not shown). Consistent with these observations, radiographical examination demonstrated that *Siglec-15*^−/−^*FcRγ*^−/−^ mice had a shorter long bone length than mice in the other groups (Fig. 2b, c). To further gain a better insight into the pathogenic mechanism of dwarfism in *Siglec-15*^−/−^*FcRγ*^−/−^ mice, radiographic and histological analyses were performed on the growth plate and primary spongiosa of the distal femur. Remarkably, a sclerotic region at the distal metaphysis close to the growth plate was observed in *Siglec-15*^−/−^*FcRγ*^−/−^ mice (Fig. 2d). Histologically, the sclerotic metaphyseal regions observed in *Siglec-15*^−/−^*FcRγ*^−/−^ mice comprised of Safranin O-positive calcified cartilage, which is normally resorbed and replaced by bone (Fig. 2e). Moreover, there were significantly fewer TRAP- and matrix metalloproteinases (MMP)9-positive cells, and more cartilage remnants in the primary spongiosa of *Siglec-15*^−/−^*FcRγ*^−/−^ mice than in mice of the other groups (Fig. 2e, f). This implies that Siglec-15 and FcRγ can complement each other during osteoclast development in primary spongiosa. These collective results indicate that double deficiency of *Siglec-15* and *FcRγ* leads to dwarfism due to dysfunction of the growth plate and impaired skeletal growth. This results from the defects in osteoclast differentiation and insufficient replacement of calcified cartilage by bone.

### Siglec-15 plays a central role in osteoclast cytoskeletal organization

**Fig. 3:**
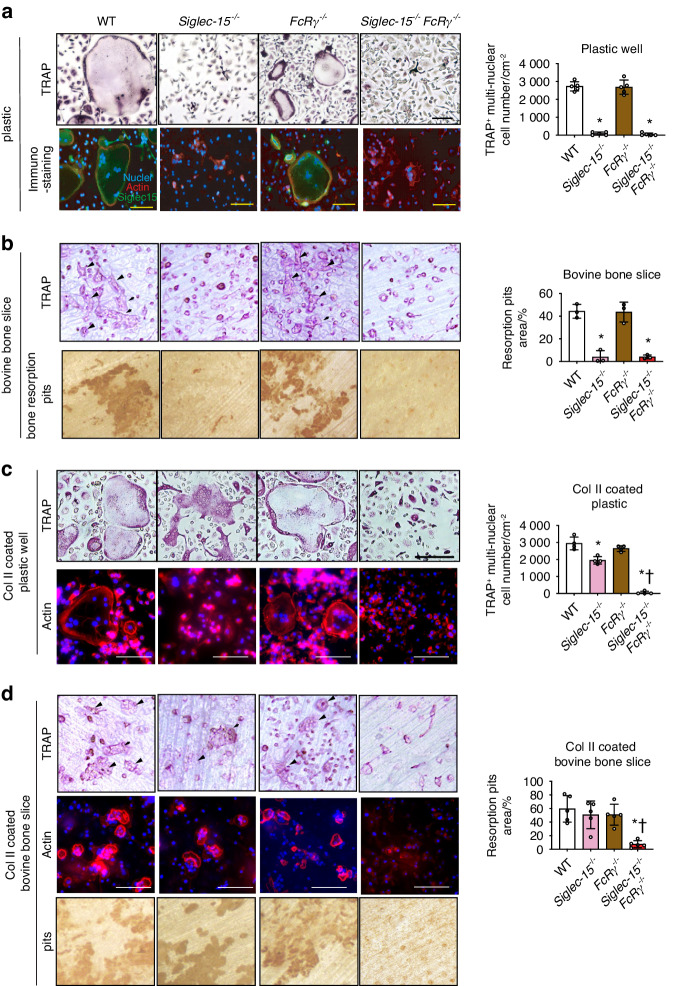
Deficiency of *Siglec-15* leads to impairment in formation of functional osteoclasts. **a** Number of tartrate-resistant acid phosphatase (TRAP)-positive multinuclear cells in bone marrow macrophage cultures of *Siglec-15*^−/−^ and Siglec*-15*^−/−^*FcRγ*^−/−^ stimulated with macrophage colony-stimulating factor (M-CSF) and receptor activator of nuclear factor κB ligand (RANKL) for 5 days on plastic plates. The left panel displays representative images of TRAP-stained cells. The right panel shows quantitation of TRAP-positive cells. Scale bars = 50μm. Immunostaining of cells by specific markers including Siglec-15 antibody (green) and fluorescently labeled phalloidin (Red). Blue indicates 4′,6-diamidino-2-phenylindole (DAPI). Scale bars = 50 μm. **b** In vitro bone resorption assay of cultured osteoclasts on bovine bone slices. Left panels represent images of stained bone slices to observe osteoclasts and pits. Scale bars = 50 μm. The right panels show percentage of quantified bone resorption pits. Scale bars = 50 μm. **c** Number of TRAP-positive cells of bone marrow macrophage cultures stimulated by M-CSF and RANKL for 5 days on collagen coated plates. The left panel displays representative images of TRAP- and phalloidin/DAPI- stained cells. The right panel shows quantification of TRAP-positive cells. **d** In vitro bone resorption assay of cultured osteoclasts on type 2 collagen-coated bovine bone slices. Left panels represent images of phalloidin/DAPI stained bone slices to observe osteoclasts and pits. Scale bars = 50 μm. The right panels show percentage of quantified bone resorption pits. Scale bars = 50 μm. Data are shown as the mean (*n* = 3) ± S.D. *P < 0.05 compared with WT mice. †*P* < 0.05 compared with *Siglec-15*^−/−^ mice

To gain additional evidence on the involvement of Siglec-15 and FcRγ in osteoclast development and maturation, bone marrow-derived macrophages (BMMs) were obtained from different mice strains and their ability to form osteoclasts and resorb bone was evaluated in vitro. TRAP-positive cells were formed from BMMs of all mice strains in the presence of M-CSF and RANKL, most of them were mononuclear cells, and the number of multinucleated cells derived from *Siglec-15*^−/−^ and *Siglec-15*^−/−^*FcRγ*^−/−^ animals was significantly lower than that derived from WT cells (Fig. 3a). Likewise, the resorption pit area on bovine bone slices after ten days of culture with M-CSF and RANKL were significantly smaller in *Siglec-15*^−/−^ cells and *Siglec-15*^−/−^*FcRγ*^−/−^ cells than in WT cells (Fig. 3b).

To explore the mechanism of defective osteoclastogenesis in primary spongiosa of *Siglec-15*^−/−^*FcRγ*^−/−^ mice, we compared the abilities of BMMs from WT, *Siglec-15*^−/−^*, FcRγ*^−/−^, and *Siglec-15*^−/−^*FcRγ*^−/−^ to form bone resorbing osteoclasts (Fig. 3c). Given the fact that type II collagen partly rescues defective osteoclastogenesis of *Siglec-15*^−/−^ cells via an osteoclast-associated collagen receptor (OSCAR)/FcRγ-mediated signaling,^7^ we performed this comparasion in the presence of type II collagen. As expected, type II collagen rescued multinuclear giant cell formation in *Siglec-15*^−/−^ BMMs, but not in *Siglec-15*^−/−^*FcRγ*^−/−^ BMMs. *Siglec-15*^−/−^ BMMs failed to form podosome belts when cultured on type II collagen-coated plastic wells but formed actin rings and restored bone resorptive activity when cultured on type II collagen-coated bovine bone slices. In contrast, *Siglec-15*^−/−^*FcRγ*^−/−^ BMMs showed no recovery of bone resorptive activity as well as actin ring formation when cultured on type II collagen-coated bovine bone slices (Fig. 3d).

### Siglec-15 may mediate cell adhesion via activating Rap1 signaling during osteoclastogenesis

**Fig. 4:**
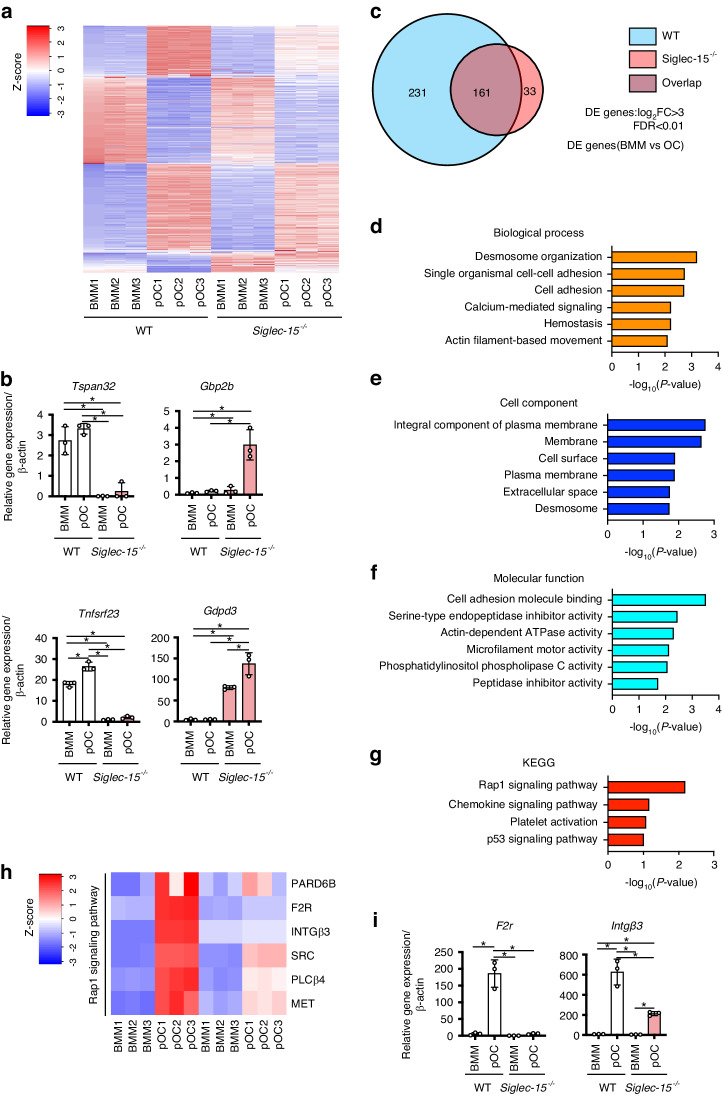
Gene expression analysis of preosteoclasts (pOC) of WT and *Siglec-15*^−/−^ mice analyzed by RNA-sequencing. **a** Heatmap for genes that are differentially expressed between BMM and pOC, *n* = 3 per group. Red and blue colors represent high and low expression levels, respectively. Each column represents a single culture, and each row represents a gene. **b** Upregulated and downregulated genes associated with osteoclast differentiation further validated the expression of BMMs and pOCs in WT and *Siglec-15*^−/−^ mice by RT-qPCR. **c** A Venn diagram indicating overlap of differential expression (DE) genes (*P* < 0.01, log_2_ (foldchange) > 3) during in vitro osteoclastogenesis between WT cells and *Siglec-15*^−/−^ cells. **d**–**g** Gene ontology (GO) analysis of genes upregulated by M-CSF and RANKL stimulation in WT but not in *Siglec-15*^−/−^ cells. The top 6 GO terms of biological process (**d**), cell component (**e**), and molecular function (**f**). **g** The KEGG pathway analysis of genes upregulated by M-CSF and RANKL stimulation in WT but not in *Siglec-15*^−/−^ cells. The top 4 pathways associated with these genes are shown. The value of −log_10_ (*P* value) was calculated to reflect the significance of GO or pathway terms. **h** Heatmap for genes involved RAP1 signaling of preosteoclasts (pOC) of WT and *Siglec-15*^−/−^ mice. **i** The expression of a group of genes related to Rap1 signaling by real-time PCR

Our finding underlining the potential involvement of *Siglec-15* in cytoskeletal organization of osteoclasts motivated us to further explore the molecular mechanism by which Siglec-15 regulates this process. The BMMs and pre-osteoclasts (pOC) of WT and *Siglec-15*^−/−^ mice were harvested and subjected to RNA-seq analysis. The differentially expressed genes (DEGs) between BMMs and pOC (false discovery rate [FDR] < 0.01) with fold change > 3 were set for further bioinformatic analyses that identified 392 genes in pOC of WT mice and 194 genes of *Siglec-15*^−/−^ mice (Fig. 4a, b). Venn analysis for DEGs showed that 161 genes were present in pOC from the WT and *Siglec-15*^−/−^ of mice, 231 genes were present in pOC of WT and 33 genes from *Siglec-15*^−/−^mice (Fig. 4c). To further understand the molecular regulatory mechanism of Siglec-15 in cytoskeletal organization of osteoclasts, the identified 231 genes present in pOC of WT but not *Siglec-15*^−/−^mice were subjected to gene ontology (GO) and pathway enrichment analyses. The top enriched terms included desmosome organization (GO:0002934, *P* < 0.001), and cell adhesion (GO:0007155, *P* < 0.01) for biological process, integral component of plasma membrane (GO:0005887, *P* < 0.01) for cell component, and cell adhesion molecule binding (GO:0050839, *P* < 0.001) for molecular function (Fig. 4d–f). Furthermore, a Kyoto encyclopedia of genes and genomes (KEGG) pathway database mapping analysis revealed that these DEGs were the most significantly enriched in the Ras-associated protein-1 (RAP1) signaling pathway term (Fig. 4g). It is noteworthy that the expression of genes enriched in cell adhesion and the RAP1 pathway in pOC of WT were higher than those in pOC of *Siglec-15*^−/−^mice (Fig. 4h, i).

**Fig. 5:**
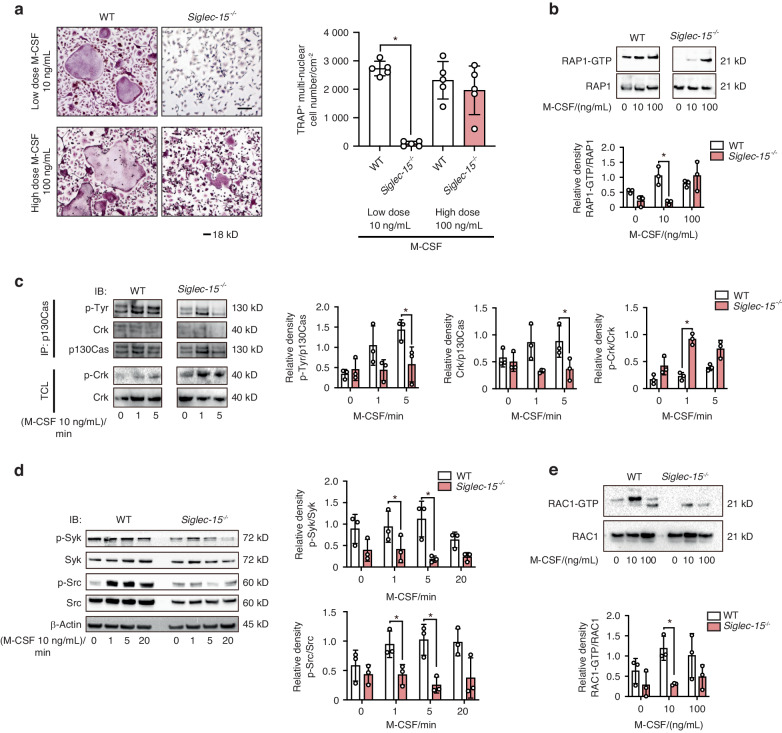
Siglec-15 is involved in M-CSF-induced activation of RAP-1 signaling. **a** *Siglec-15*^−/−^ cells do not form osteoclasts upon low-dose M-CSF stimulation but are partially rescued and form osteoclasts upon high-dose M-CSF stimulation. Osteoclasts were generated from bone marrow macrophages of WT and *Siglec-15*^−/−^ mice cultured for 5 days with RANKL (100 ng/mL) and low (10 ng/mL) or high (100 ng/mL) dose M-CSF. Left panel shows osteoclasts stained by TRAP staining. The right panel shows quantitation of TRAP-positive cells with at least three nuclei. Scale bars = 50 μm. *P < 0.05 compared with WT cells by paired *t*-test. **b**, **e** WT and *Siglec-15*^−/−^ prefusion osteoclasts were starved for 1 h in α minimum essential medium (MEM) and then stimulated with 0, 10, or 100 ng/mL M-CSF. The signals were quantified by densitometry; *P < 0.05 as determined by paired *t*-test. **c**, **d** Pre-fusion osteoclasts were starved for 1 h in αMEM and then stimulated with 10 ng/mL M-CSF for 0, 1, 5, or 20 min. The signals were quantified by densitometry; *P < 0.05 as determined by paired *t*-test. (**c**)Syk and Src phosphorylation was detected by immunoblot. GTP-RAP1 (**b**) and GTP-RAC1 (**e**) in the lysates were measured by pulldown assay. The signals were quantified by densitometry; *P < 0.05 as determined by paired *t*-test. Data are shown as the mean ± SD

Considering these collective results, we conclude that Siglec-15 mediates cell adhesion and RAP1 pathway activation that are essential for cytoskeletal organization and maturation of osteoclasts. These results, together with the facts that RAP1 is the downstream molecule activated by M-CSF and that Siglec-15 deficiency has a slight impact on calcium signaling and induction of NFATc1 during RANKL-induced osteoclastogenesis (Fig. S2), prompted us to speculate that the Siglec-15/DAP12 pathway facilitates M-CSF induced signaling for cytoskeletal organization in osteoclasts. To verify our speculation, we first examined the effects of different doses of M-CSF on the activation of RAP1 in BMMs referring to reports that defective cytoskeletal reorganization in *DAP12*^−/−^ osteoclasts are partially rescued by high-dose M-CSF.^3,18^ Of note, high dosage of M-CSF rescued defective cytoskeletal reorganization in *Siglec-15*^−/−^ cells, which showed increased expression of an active GTP-bound RAP1 form comparable to that of WT cells. In contrast, minimum dosage of M-CSF that is required for osteoclast differentiation and RAP1 activation was not effctive in Siglec-15 deficient preosteoclasts (Fig. 5a, b). To further gain additional evidence on the involvement of Siglec-15 in activation of RAP1 signaling, we investigated the expression of upstream regulators of RAP1, including p130Cas and CrkII.^19,20^ The phosphorylation of p130Cas and the association of p130Cas and CrkII in response to M-CSF stimulation were impaired in *Siglec-15*^*−/−*^ cells (Fig. 5c). In contrast, these cells exhibited an increase in the phosphorylation of CrkII, which inactives CrkII. Active RAP1 promotes transition of the αvβ3 integrin to its high-affinity conformation (inside-out integrin activation). This further leads to the activation of canonical integrin-signaling consisting of c-Src, Syk, and Ras-related C3 botulinum toxin substrate 1 (RAC1). Given these facts, we tested whether Siglec-15 is involved in canonical integrin signaling induced by M-CSF and is associated with activation of c-Src, Syk, and RAC1. Interestingly, we found that phosphorylation of c-Src and Syk due to M-CSF stimulation was impaired in *Siglec-15*^−/−^ preosteoclasts compared to WT preosteoclasts (Fig. 5d). Furthermore, activation of RAC1, which is the currently established most distal component of a canonical osteoclast cytoskeletal organizing complex, was also impaired in *Siglec-15*^−/−^ preosteoclasts compared to that of WT preosteoclasts (Fig. 5e). These collective results revealed that Siglec-15 is a critical DAR for physiological bone remodeling involving in M-CSF-induced activation of the RAP1/RAC1 pathway which is essential for formation and maturation of osteoclasts.

## Discussion

The aim of the current study was directed to answer an important question in bone biology and clarify the most critical DAR mediating the formation of functional osteoclast and physiological bone resorption. To this end, *Siglec-15*^−/−^, *Trem-2*^−/−^, *Clec5a*^−/−^, *FcRγ*^−/−^*, Siglec-15*^−/−^*FcRγ*^−/−^*, Trem-2*^−/−^*FcRγ*^−/−^, and *Clec5a*^−/−^*FcRγ*^−/−^ mice were generated and their bone mass parameters in the femur were evaluated. Doubly deficient mice in *FcRγ* and DARs were used to rule out the possibility that FcRγ signaling compensates for the absence of DAR/DAP12 signaling. Our results revealed that Siglec-15 is a critical DAR that regulates cytoskeletal organization of osteoclasts and their physiological bone resorption.

*Trem-2*^−/−^, *Clec5a*^−/−^, *FcRγ*^−/−^*, Trem-2*^−/−^*FcRγ*^−/−^, and *Clec5a*^−/−^*FcRγ*^−/−^ mice exhibited normal bone phenotype in contrast to *Siglec-15*^−/−^ and *Siglec-15*^−/−^*FcRγ*^−/−^ mice that exhibited mild to severe osteopetrosis. Importantly, similar bone phenotype is evident in *Dap12*^−/−^*FcRγ*^−/−^ mice due to the defective cytoskeletal organization of osteoclasts and impairment of physiological bone remodeling.^2,21^ In fact, *Siglec-15*^−/−^ osteoclasts failed to spread and form an actin ring and exhibited a poor bone resorptive capacity in vitro, in analogous fashion to *Dap12*^−/−^ osteoclasts.^6,10^ These findings underline the critical role of Siglec-15/DAP12 signaling in skeletal development as well as physiological bone remodeling. However, the differences in bone phenotypes observed in *Siglec-15*^−/−^*FcRγ*^−/−^ mice, but not in *Siglec-15*^−/−^ or *FcRγ*^−/−^ mice can be explained based on the fact that osteoclast-associated receptor (OSCAR) is an FcRγ associated receptor,^22,23^ and its recognition of the collagen motifs rescues *Dap12*^−/−^ cells to form osteoclast-like cells.^7,24^ In line with the in vivo findings, we noted that type II collagen, the primary component of the cartilage matrix and abundant in primary spongiosa, rescues the failure of *Siglec-15*^−/−^ cells, but not *Siglec-15*^−/−^*FcRγ*^−/−^ cells to form multinucleated osteoclasts. Morover, growing rats treated with anti-Siglec-15 neutralizing antibodies exhibit normal skeletal growth due to the preservation of osteoclast differentiation in primary spongiosa, whereas those treated with alendronate exhibited impaired skeletal growth due to defective osteoclasts in the primary spongiosa.^25^ Siglec-15 appears to be involved only in physiological bone resorption since *Siglec-15*^−/−^ mice do not suppress bone erosion in a murine model of inflammatory arthritis.^26^ Another important finding in our study is that *Siglec-15*^−/−^*FcRγ*^−/−^ mice exhibited skeletal growth failure and dwarfism features due to defective absorption of calcified cartilage in the growth plate. It is important to mention that *Dap12*^−/−^*FcRγ*^−/−^ mice exhibit dwarfism features similar to *Siglec-15*^−/−^*FcRγ*^−/−^ mice.^3^ These collective findings indicate that the differentiation of chondroclasts or modeling osteoclasts requires Siglec-15/DAP12 and OSCAR/FcRγ signaling that together orchestrate differentiation and maturation of functional osteoclasts.

Cells lacking *Siglec-15* differentiate to osteoclasts, but the resulting cells failed to spread and form an actin ring and exhibited a poor bone resorptive capacity in vitro. Siglec-15 seemed to be involved in M-CSF induced signaling for cytoskeletal organization through RAP1/RAC1 activation, without affecting downstream osteoclastogenic transcription factors of RANKL signaling.^7^ Cells from *Siglec-15*^−/−^ mice exhibited lower expression of genes enriched in the RAP1 pathway and cell adhesion than those in WT mice. Therefore, defective cytoskeletal remodeling in *Siglec-15*^−/−^ mice might be due to impaired M-CSF-induced signaling pathway required for maturation of functional osteoclasts. This phenomenon has been documented in mice deficient in *Dap12* and *Itgb3*, which suggests that these molecules cooperatively work to organize cytoskeletal remodeling of osteoclasts through the M-CSF signaling.^21^ It is well documented that M-CSF signaling, in concert with integrins, regulates cytoskeletal organization of osteoclasts via activation of c-Fms signaling,^27^ that turns RAP-1 to its active, GTP-bound form, resulting in transition of αvβ3 integrin to its high-affinity ligand-binding conformation (inside-out integrin activation).^28,29^ Upon integrin occupancy, c-Src activates the DAP12-Syk signaling pathway,^15^ eventually resulting in activation of RAC signaling, which is known as an essential component of the canonical osteoclast cytoskeletal organization complex. In fact, it is evident that the RAC1 pathway controls actin microfilament organization and bone resorption.^30,31^

**Fig. 6:**
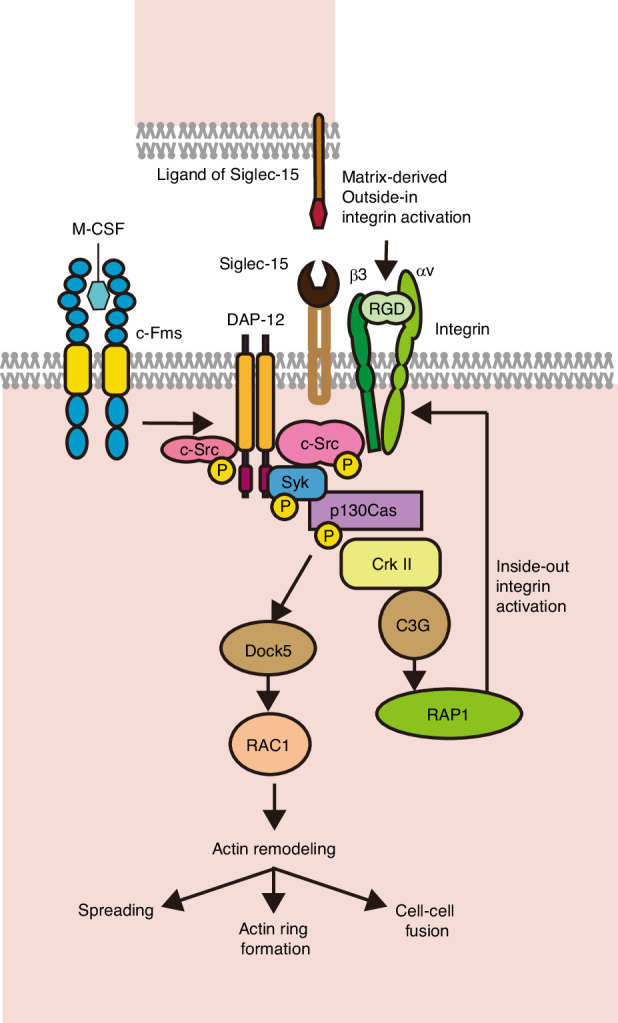
Proposed model of Siglec-15-mediated cytoskeletal regulation mechanism of osteoclasts. M-CSF signaling is required for Siglec-15-mediated activation of Rac, which is the distal component of a canonical osteoclast cytoskeletal organization complex. Siglec-15 promotes the recruitment of Syk/p130Cas complex to Dap12, thereby enhancing c-Src/p130Cas/CrkII pathway

Our data demonstrated that Siglec-15 can promote M-CSF-induced activation of the c-Src/p130Cas/CrkII pathway,^19,20,32^ which in turn activate RAP-1 and form GTP-bound, probably via a guanidine exchanging factor C3G (Fig. 6). The impairment of c-Src phosphorylation induced by M-CSF in *Siglec-15* deficient mice suggests that Siglec-15 facilitates c-Src activation as a consequence of M-CSF-stimulated inside-out integrin activation. Given that DAP-12-mediated M-CSF signaling promotes proliferation in macrophages,^33^ Siglec-15 has complementary function in the cytoskeletal organization of osteoclasts shifting the role of M-CSF signaling from proliferation to differentiation of osteoclasts.

Our data using knockout mice showed that neither TREM-2 nor CLEC5A play a role in physiological bone resorption. Although, it is evident that these immunoreceptors mediate osteoclast differentiation and maturation in pathological conditions such as inflammation. In fact, TREM-2 is the first DAR that was examined in osteoclasts, but its function in the bone environment has not reached a consensus due to its enigmatic functions. The functional deficiency of TREM-2 in humans was associated with Nasu-Hakola disease, which is characterized by rapidly progressive dementia with large bony cysts and osteopenia. Moreover, human TREM-2 deficient osteoclast precursors failed to differentiate to mature osteoclasts in vitro.^9,10^ Humphrey et al. reported that blockade of TREM-2 in mature osteoclasts inhibits bone resorption activity and silencing this molecule suppresses osteoclastogenesis in RAW264.7 cells.^9^ In contrast, a recent study by Otero et al. demonstrated that TREM-2 deficiency in mice resulted in reduced osteoclast precursor proliferation but accelerated osteoclastogenesis in vitro, leading to osteopenia in vivo.^34^ Moreover, there are no reports showing that TREM-2-deficient mice exhibit a bone phenotype similar to that of Dap12-deficient mice. These findings together indicate that TREM-2 is not a DAR that mediates the activation signal required to control physiological bone resorption. On the other hand, CLEC5A does not contribute to physiological bone resorption, but plays a critical role in development of inflammatory bone loss in inflammatory arthritis.^12^ This seems to be mediated by the CLEC5A-DAP12 axis that promotes osteoclast development and function via amplifying NFATc1, the master transcription factor for osteoclast differentiation in vitro.^11^ These collective findings suggest that TREM-2 and CLEC5A act as immunoreceptors regulating bone remodeling in specific pathological conditions. Therefore, further research, including a study on the involvement of TREM-2 and CLEC5A in bone diseases such as osteomyelitis, bone tumor, and fracture healing will be necessary.

The major limitation of this study is that we investigated the role of only three major DARs of at least 13 members present in myeloid cells^4,5^ in cytoskeletal remodeling. However, although the possible involvement of other DARs in physiological bone remodeling and skeletal development cannot be ruled out, our data provided strong evidence on fundamental role of Siglec-15 in physiological bone remodeling.

## Materials and methods

### Generation of knockout mice

We conducted all animal studies following protocols approved by the institutional committee on animal resources (Approval No. # 20-0062). All mice strains used in the study had a C57BL/6 background and were maintained under specific pathogen-free conditions. *Siglec-15*^−/−^ mice were generated by homologous recombination as previously described.^7^ *FcRγ*^−/−^ mice generated by homologous recombination^35^ were obtained from Dr. Takai (Tohoku University). *Trem-2*^−/−^ and *Clec5a*^−/−^ mice were generated using the CRISPR/Cas9 gene-editing technique (Fig. S3). The guide RNA (gRNA) was briefly inserted into the pX330-U6-Chimeric BB-CBh-hSpCas9 plasmid vector using the BbsI restriction enzyme site, and the vectors were injected into the pronuclei of pronuclear-stage embryos of C57BL/6 J female mice. These embryos were transferred into foster mothers, and then the male and female founders carrying a heterozygous (+/−) deletion mutation were inbred to produce homozygous (−/−) mice. The design of the pX330-guide and injection, followed by the transfer of embryos, was performed at the Laboratory Animal Resource Center at the University of Tsukuba (https://www.md.tsukuba.ac.jp/LabAnimalResCNT/public_E/index.html). DAR/*FcRγ* double knockout mice were obtained by crossing each DAR-deficient strain with the *FcRγ*-deficient strain. The presence of mutations in knockout mice was confirmed by PCR genotyping and sequence analysis using primer sequence sets (Table S1). Body length and weight the deficient mice were determined longitudinally for a period of one year starting at 10 weeks of age.

### X-ray imaging and BMD measurement

X-ray imaging and DXA of the 14-week-old male mice were performed by Kureha Special Laboratory Co. (Fukushima, Japan). The left femur BMD (mg/cm^2^) was calculated from the bone mineral content (mg) and bone area (cm^2^). Since Siglec-15-deficient mice showed a mild osteopetrosis phenotype in females similar to that in males,^36^ the bone phenotype was basically analyzed using male mice in this study.

### Micro-CT analysis

The left femurs of the 14-week-old male mice were scanned by micro-CT (R_mCT2; Rigaku, Japan) at a 10 μm isotropic resolution. One hundred slices covering a 1 000-μm area of interest and encompassing the distal metaphysis region, which started from 300 μm proximal to the growth plate, were used to assess bone morphology. Measures of trabecular bone and cortical bone parameters, including trabecular BV/TV, Tb.Th, Tb.N, Tb.Sp, and cortical bone thickness (Ct.th), were performed using TRI/3D-BON software (Ratoc System Engineering Co., Japan) according to the manufacturer’s instructions.^37^

### Histology and histomorphometry

The distal femur of 14-week-old male mice was fixed in paraformaldehyde, decalcified with 5% ethylenediaminetetraacetic acid disodium salt, and embedded in paraffin. Sections with 3–5 µm were stained by TRAP staining kit and methyl green (Vector Laboratories, Ontario, Canada) as counterstain to observe osteoclasts. Observation and imaging were caried out using a BX53 microscope (Olympus, Tokyo, Japan). For dynamic bone histomorphometry, tetracycline (25 mg/kg, Sigma-Aldrich, MP, USA) and calcein (20 mg/kg; Dojindo Laboratories, Kumamoto, Japan) were subcutaneously injected at 4 and 2 days before the mice were euthanized. The right tibias of 14-week-old mice were fixed in 70% ethanol and stained with Villanueva Bone Stain. The undecalcified bone sections were prepared, and histomorphometric analysis was performed blindly at the Ito Bone Histomorphometry Institute (Niigata, Japan). The number of osteoclasts on bone surface (N.Oc/BS) and Oc.S/BS at the primary and secondary spongiosa were measured using ImageJ software (National Institutes of Health, NIH, Bethesda, MD, USA). Primary spongiosa was defined as the area 250-μm proximal to the growth plate. The secondary spongiosa was defined as the area 250–1 000-μm proximal to the growth plate. Cartilage remnant at the primary spongiosa was measured as a percentage of the trabecular bone area in the sections stained with Safranin O.

### In vitro osteoclastogenesis

BMMs were harvested from the femurs and tibia of 7- to 9-week-old mice as previously reported.^38^ Briefly, the marrow cells were collected from femurs and tibias. After removing the red blood cells, marrow cells were cultured on a suspension culture dish in the presence of 50 ng/mL M‐CSF for 3 days to enrich the CD11b^+^ (Mac1^+^) population. Osteoclast differentiation from BMMs was performed as previously reported.^7,38^ The BMMs were cultured with 10 ng/mL M-CSF (PeproTech, London, UK) and 100 ng/mL RANKL (PeproTech) for five days at 37 °C in a 5% humidified CO_2_ incubator, and osteoclastogenesis was confirmed by TRAP staining using a histochemical kit (Sigma-Aldrich, MO, USA). For actin staining, cells were seeded on 48-well plates at 1 × 10^4^/well. TRAP-positive multinucleated cells with at least three nuclei were counted as multinucleated osteoclasts. The cytoskeletal actin was stained using Alexa Fluor 633 phalloidin (Invitrogen Molecular Probes) and the nuclei were stained using 4’,6‐diamidino‐2‐phenylindole reagent (Dojindo Laboratories, Kumamoto, Japan). Alexa 633 and DAPI were excited by 633 nm and 405 nm laser lines, respectively, and were visualized with a fluorescence microscope (BZ-X710; Keyence, Japan).

### In vitro resorption assay

The BMMs were cultured with M-CSF and RANKL (PeproTech) to generate osteoclasts on bovine bone slices for 10 days. Cells were removed by ultrasonic disruptor and then stained with 20 μg/mL peroxidase-conjugated wheat germ agglutinin (Sigma) and 3,3’-diaminobenzidine (0.52 mg/mL in phosphate buffered saline (PBS) containing 0.1% H_2_O_2_) (Tokyo Kasei Industries, Tokyo, Japan). Resorption pit areas were measured using ImageJ software (NIH, USA).^7^ All in vitro osteoclastogenesis and resorption assays were performed with biological replicates (3–5 technical replicates).

### RNA sequencing

The BMMs isolated from WT and deficient mice were differentiated into pOC by treatment with M-CSF and RANKL (PeproTech) for 3 days. The BMMs and pOCs of WT and *Siglec-15*^−/−^ mice were lysed with TRIzol Reagent (Thermo Fisher scientific, MA, USA), and harvested for RNA extraction. Total RNA was extracted using RNeasy Plus Mini Kit (Qiagen, Hilden, Germany) according to the manufacturer’s instructions. The integrity of samples was assessed by determining 28 S/18 S ribosomal RNA bands with an Agilent 2100 bioanalyzer (Agilent Technologies, Santa Clara, CA). High-quality libraries assayed by Bioanalyzer High sensitivity DNA kit (Agilent) were subjected to NovaSeq 6000 (Illumina, CA, USA). Sequencing yield was typically ∼25 million strand-specific reads. The obtained mRNA sequencing reads were mapped against the mouse genome (GRCm38) using STAR (Version 2.5.3a) and gene expression level was quantified using RNA-Seq by expectation-maximization (RSEM) (version 1.2.31).^39^ Differential expression analysis was performed with the edgeR package (Version 3.22.5) within the R programming environment (Version 3.5.1). Genes with FDR < 0.01 were considered to be significantly DEGs. Furthermore, GO analysis and KEGG analyses were used to investigate the roles of genes upregulated by M-CSF and RANKL stimulation in WT but not in *Siglec-15*^−/−^ cells by using the Database for Annotation Visualization and Integrated Discovery online tools (DAVID: https://david.ncifcrf.gov/). The RNA-seq data included in this study are publicly available at the Gene expression omnibus (GEO) database (https://www.ncbi.nlm.nih.gov/geo/) with an accession number GSE218768.

### Immunoblot analysis

The BMMs were cultured in the presence of 30 ng/mL M-CSF and 100 ng/mL RANKL (PeproTech). Thereafter, the cells were washed twice with ice-cold PBS and the cell lysates were extracted using the PhosphoSafe Extraction Reagent (Novagen, Madison, WI, USA) with protease inhibitor cocktail set III (Calbiochem, San Diego, CA, USA). Immunoprecipitation experiments were performed using the Dynabeads Protein A immunoprecipitation kit (Invitrogen, Carlsbad, CA, USA) according to the manufacturer’s instructions. Lysates were subjected to immunoblot or immunoprecipitation analyses using the indicated antibodies. The sources of antibodies are as follows: anti-phosphotyrosine (p-Tyr) antibody (4G10, 05-321) was purchased from Merk-Millipore (Darmstadt, Germany); anti-p130Cas (397666) and anti-Crk (397452) antibody were purchased from Becton-Dickinson biosciences (NJ, USA); Syk (#2712), phospho-CrkII (Tyr221, #3491) phospho-Syk (Tyr519/520, #2710), Src (#2108), phosphor-Src (Tyr416, #2101), and β-Actin (#4967) antibodies were purchased from Cell Signaling Technology (MA, USA). The amount of active Rac1 or Rap1 was determined by affinity precipitation using the active Rac 1 pull-down and detection kit (Thermo Fisher scientific) and Rap1 activation assay kit (Millipore, MA, USA). Densitometry analysis was performed using ImageJ software (NIH, USA) on immunoblots from three independent experiments.

### Statistical analysis

Statistically significant differences among groups were determined by one-way analysis of variance (ANOVA) followed by the Tukey test for multi-group comparison. In the longitudinal analysis, Two-way ANOVA followed by the Tukey test was used to test for significant difference. *P* < 0.05 was considered statistically significant. All data are shown as mean ± standard deviation Statistical analysis was performed using Prism software (Graph Pad Prism version 8.4.3, USA).

### Supplementary information

Comprehensive blot images

Supplementary Fig. 4

Supplementary Figure 1

Supplementary Figure 2

Supplementary Figure 3

Supplementary Table

Supplementary Figure legends

## Data Availability

The datasets generated during and/or analyzed during the current study are available in the GEO repository, https://www.ncbi.nlm.nih.gov/geo/query/acc.cgi?acc=GSE218768.
