# Supplementary figures and images for: Involvement of Siglec-15 in regulating RAP1/RAC signaling in cytoskeletal remodeling in osteoclasts mediated by macrophage colony-stimulating factor
Source: Bone Res. 2024 Jun 7;12:35. doi: 10.1038/s41413-024-00340-w (PMC11161467; doi:10.1038/s41413-024-00340-w)

P Syk

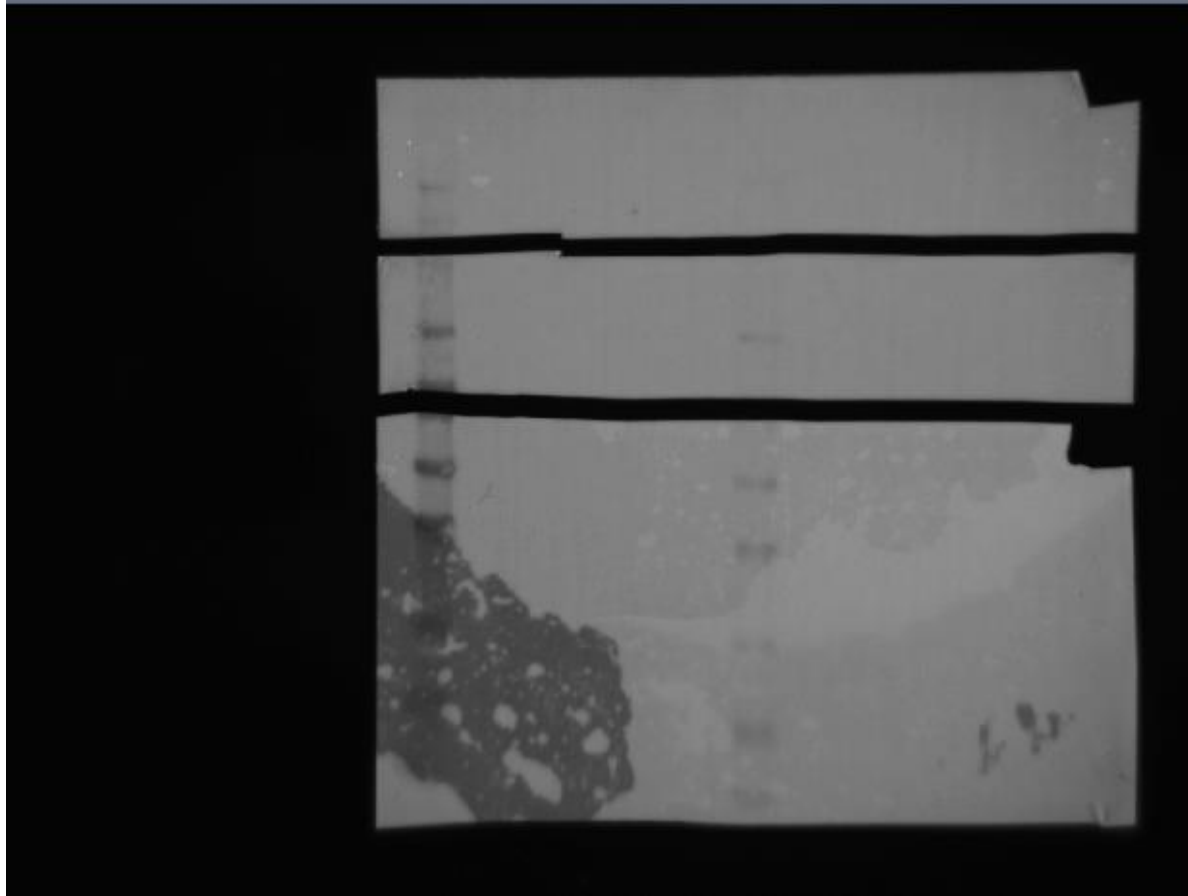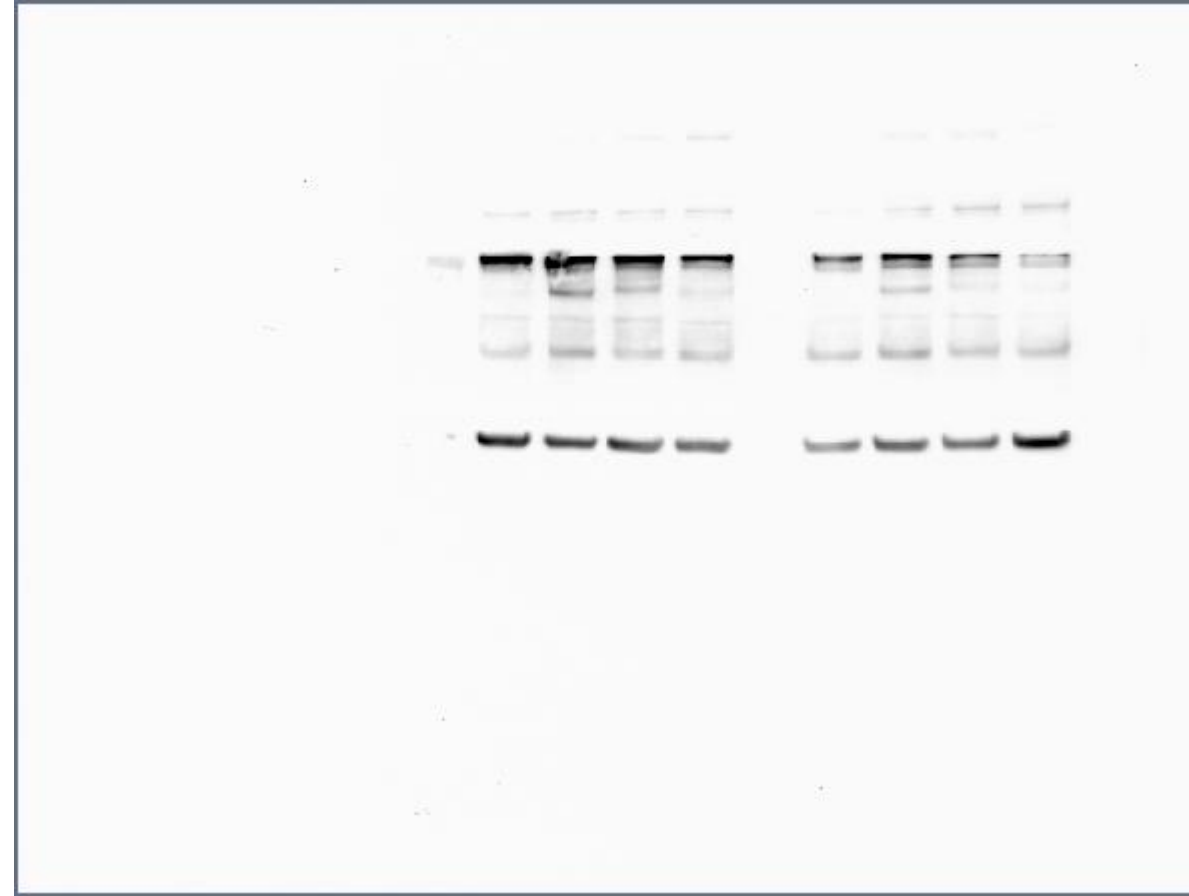

Syk

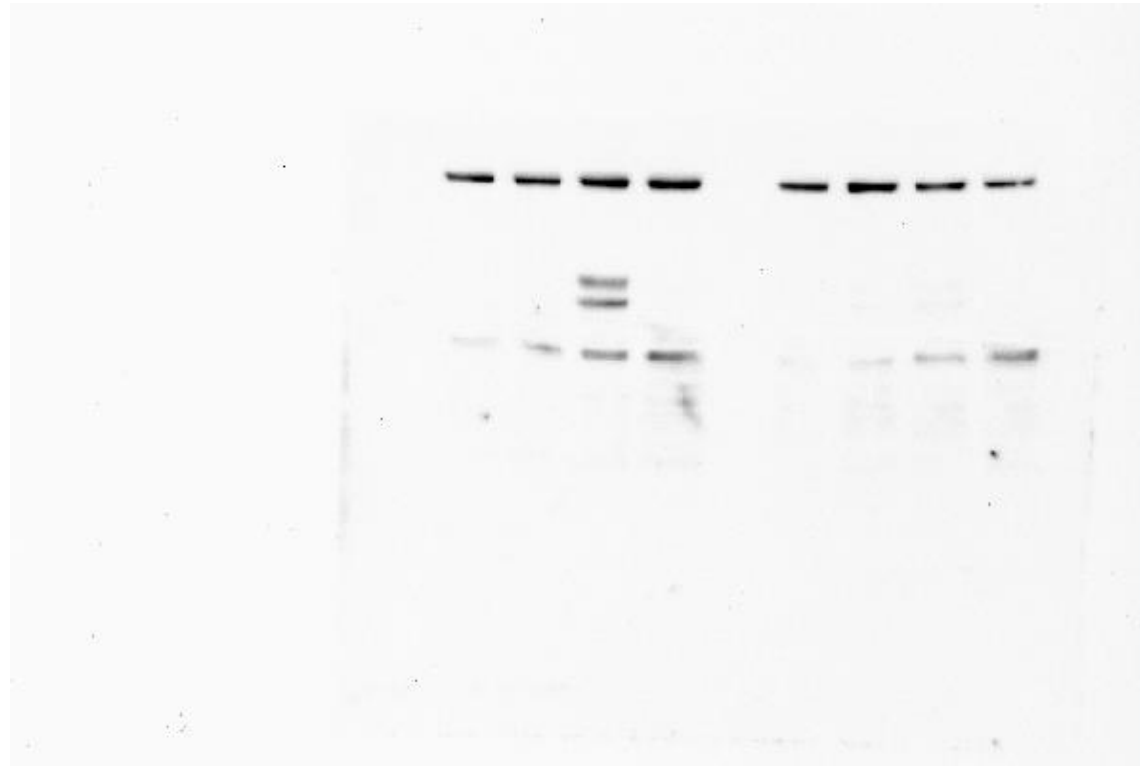

P Src

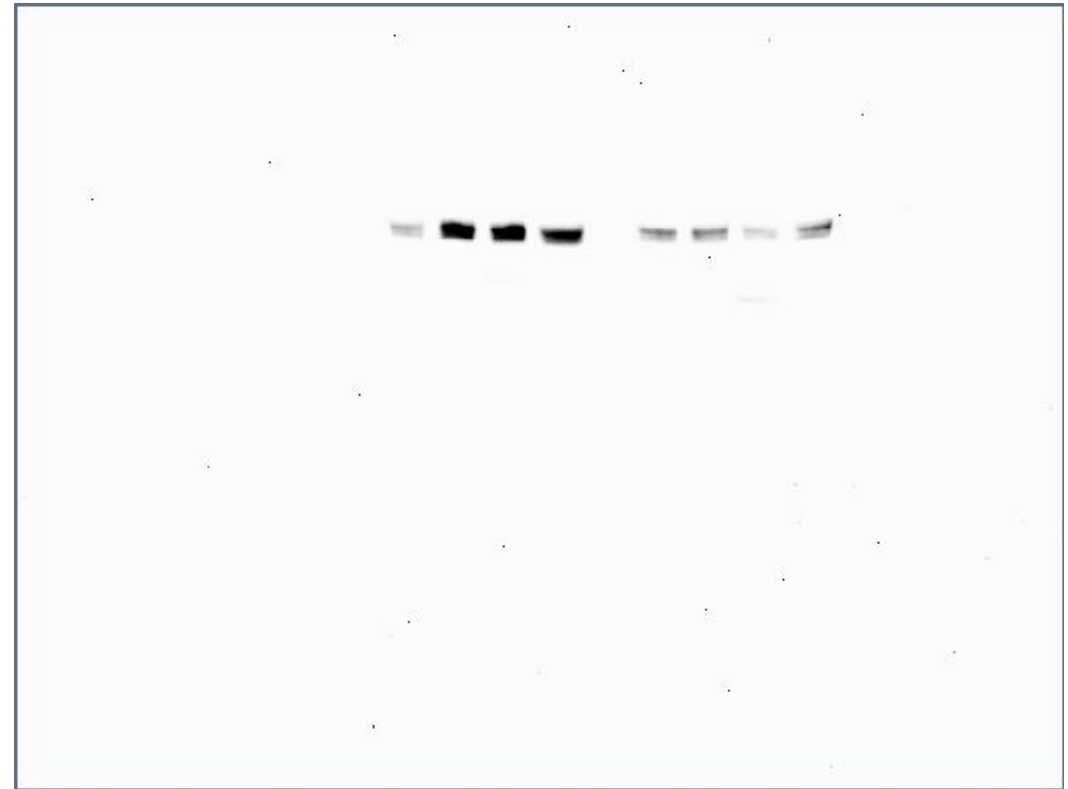

S r c

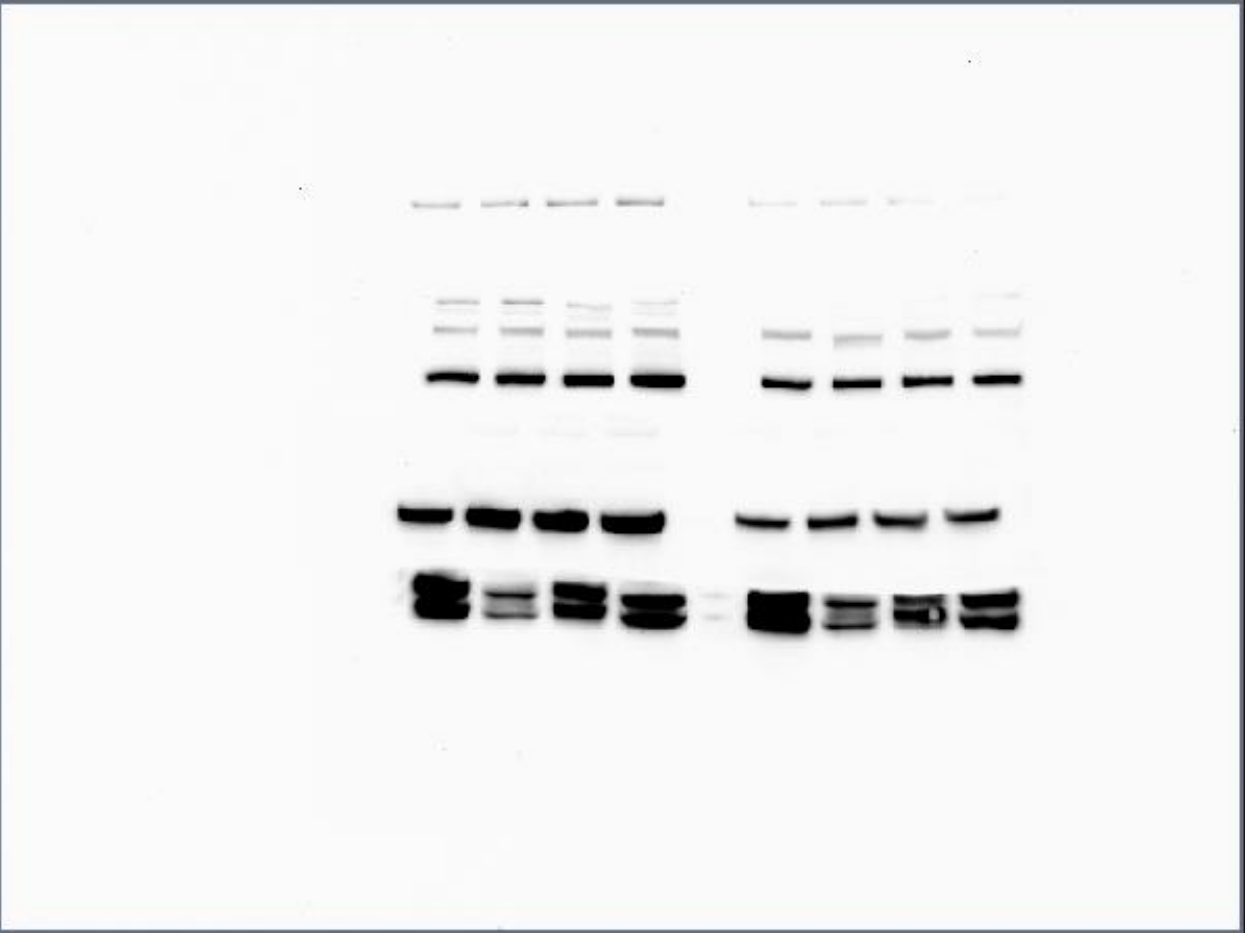

A c t i n

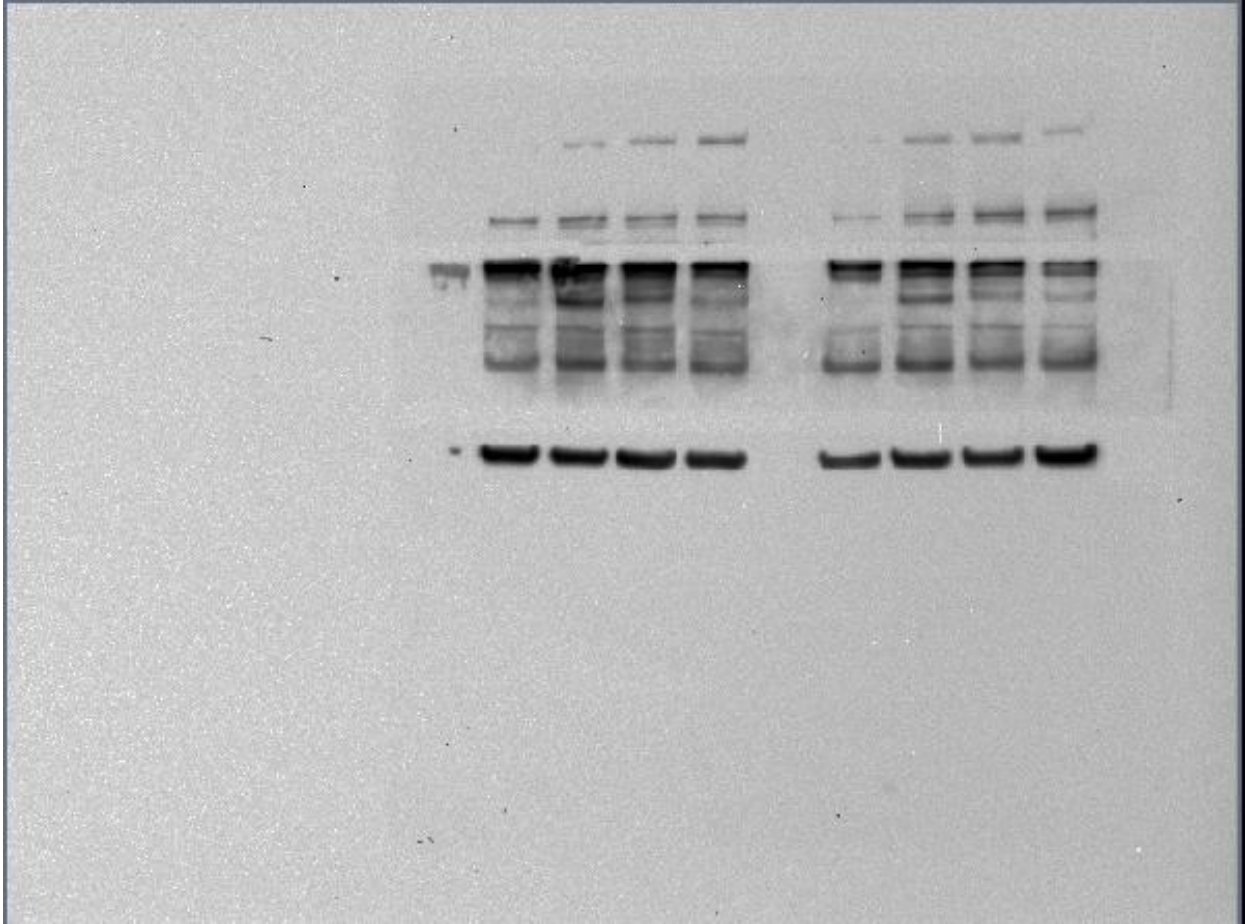

Rap1

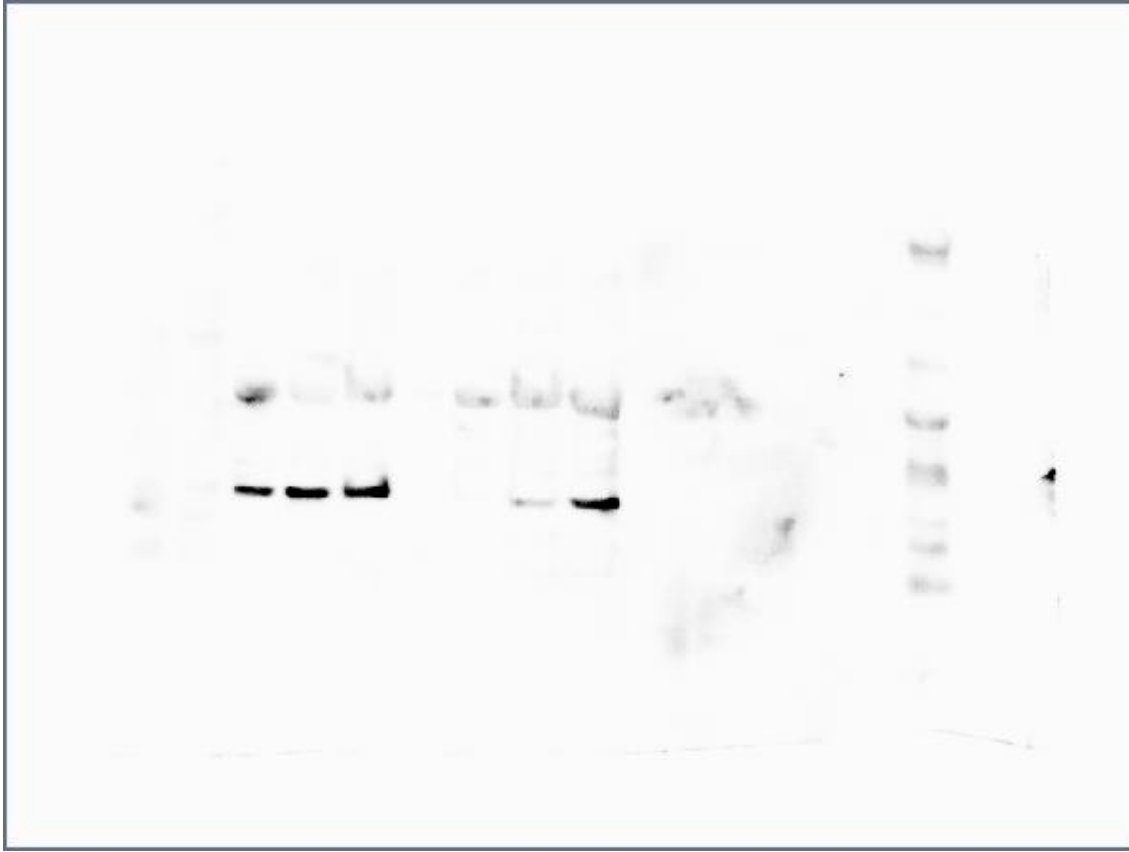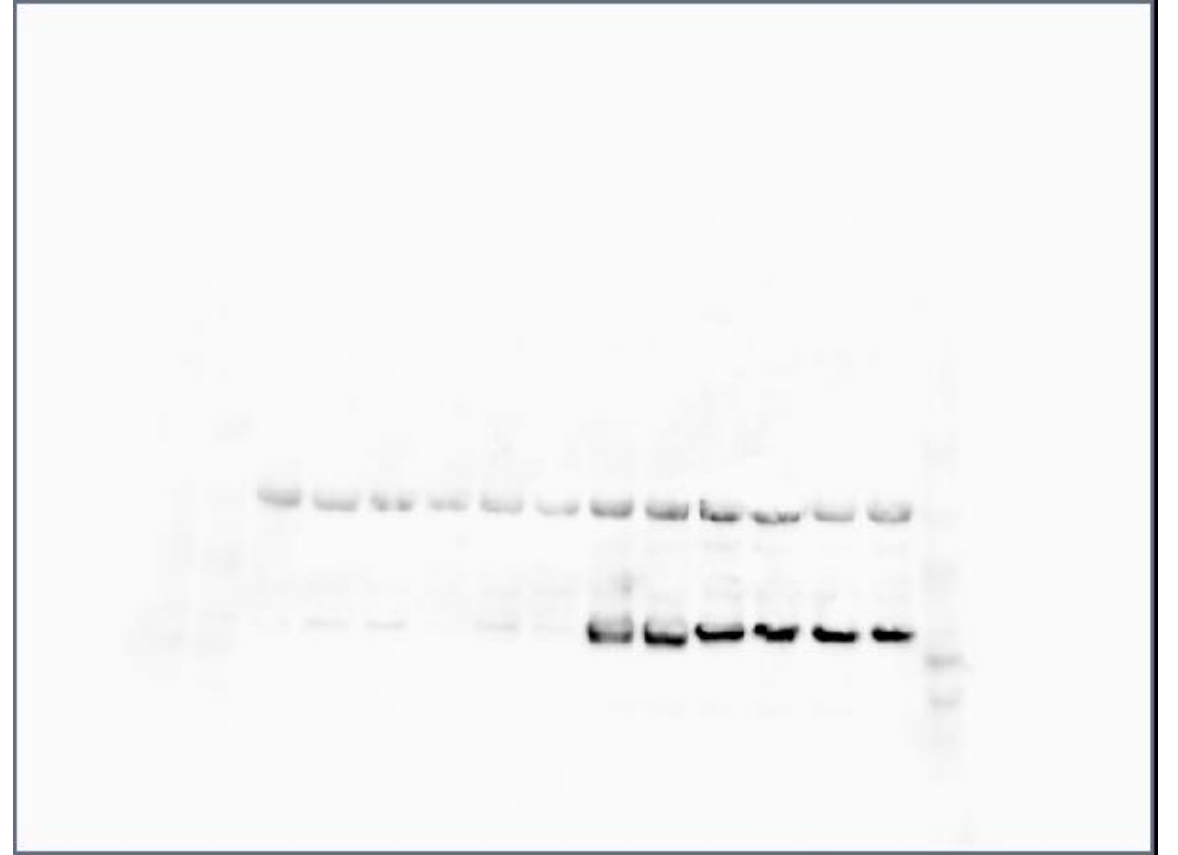

Rac1

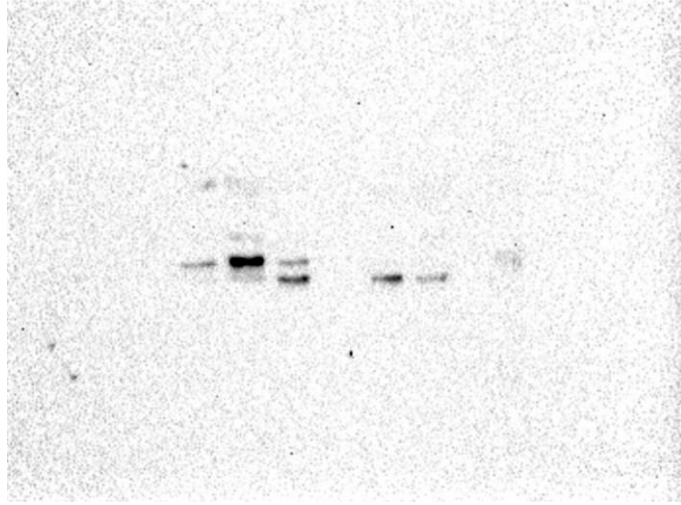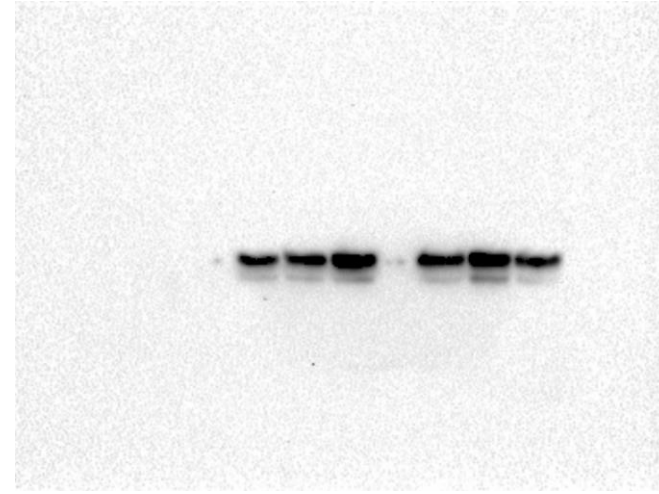

P130cas IB 4g10

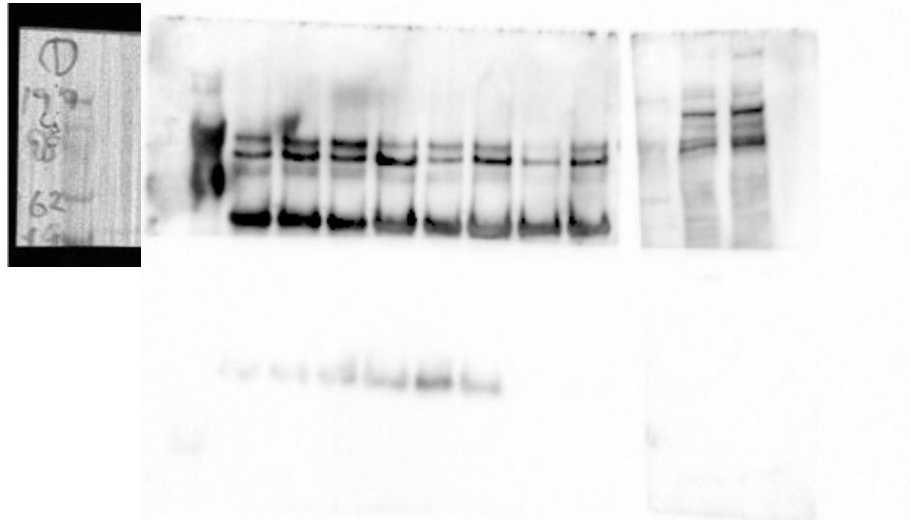

P130cas IB p130cas

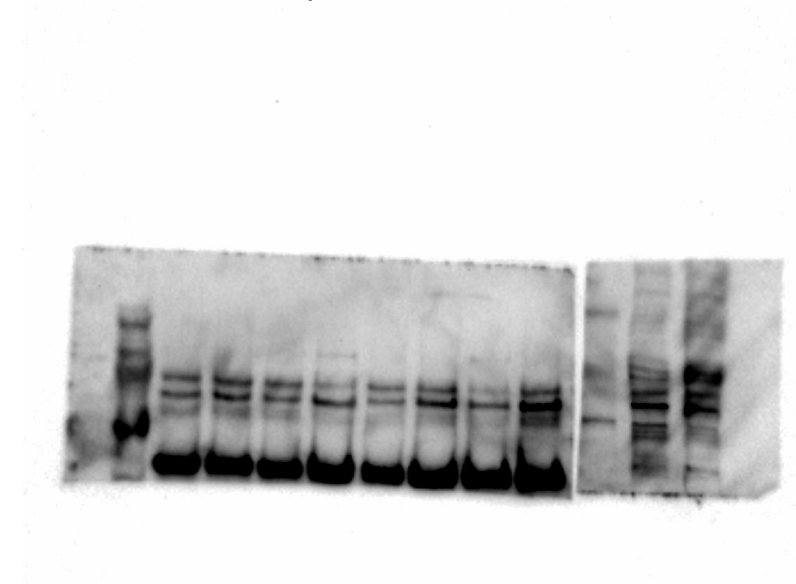

P130cas IB Crk

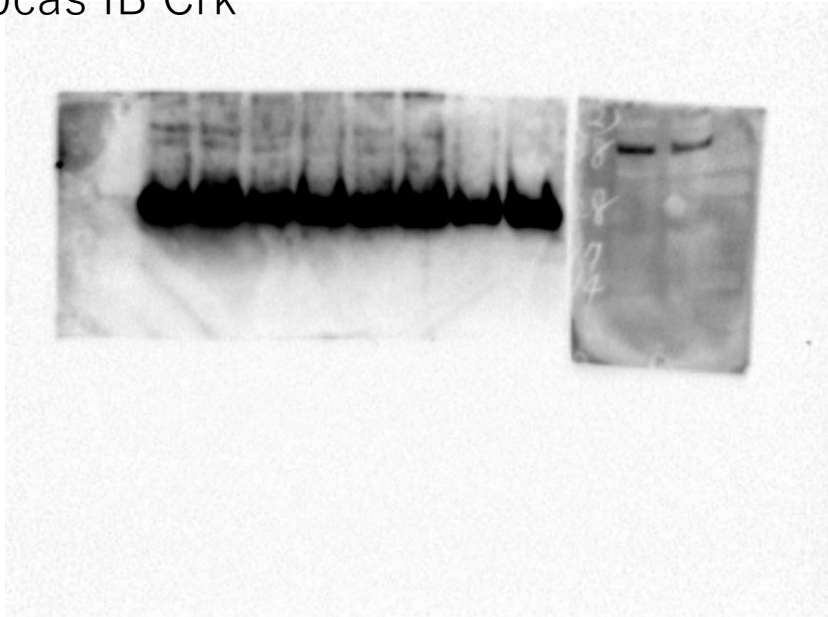

pCrk

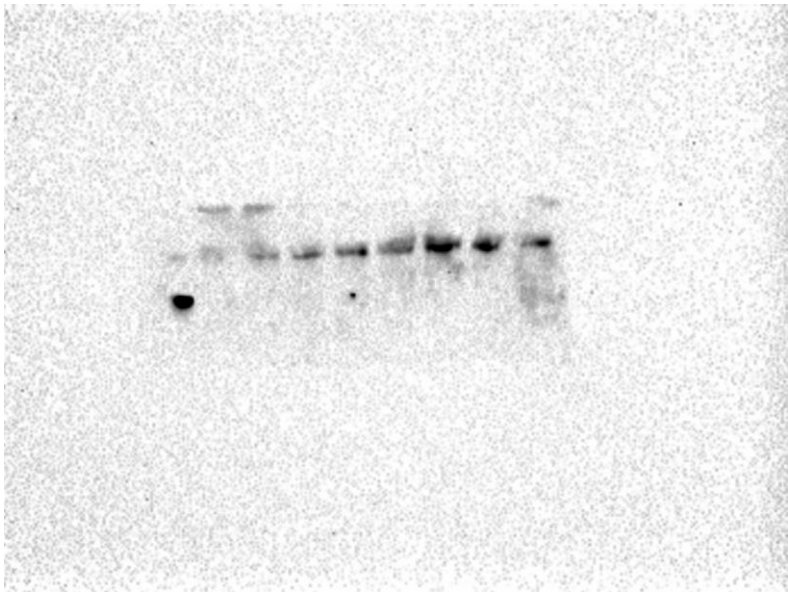

Crk

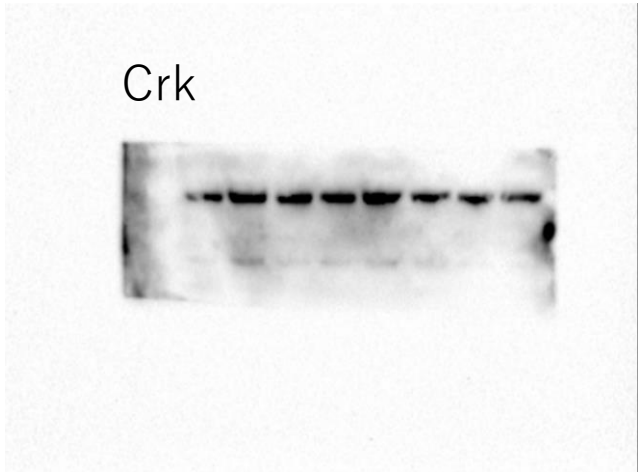

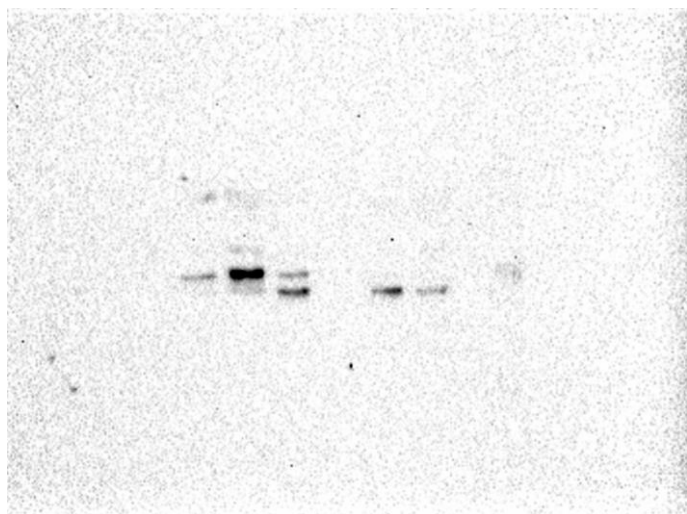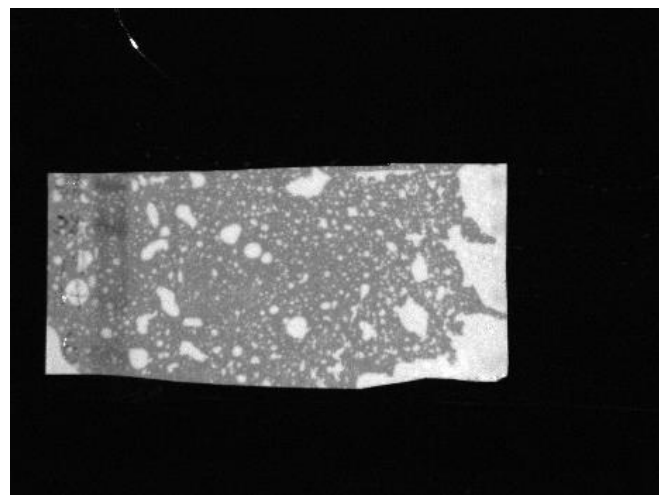

Supplement: Supplementary file 1 — Comprehensive blot images [file 41413_2024_340_MOESM1_ESM.pdf]

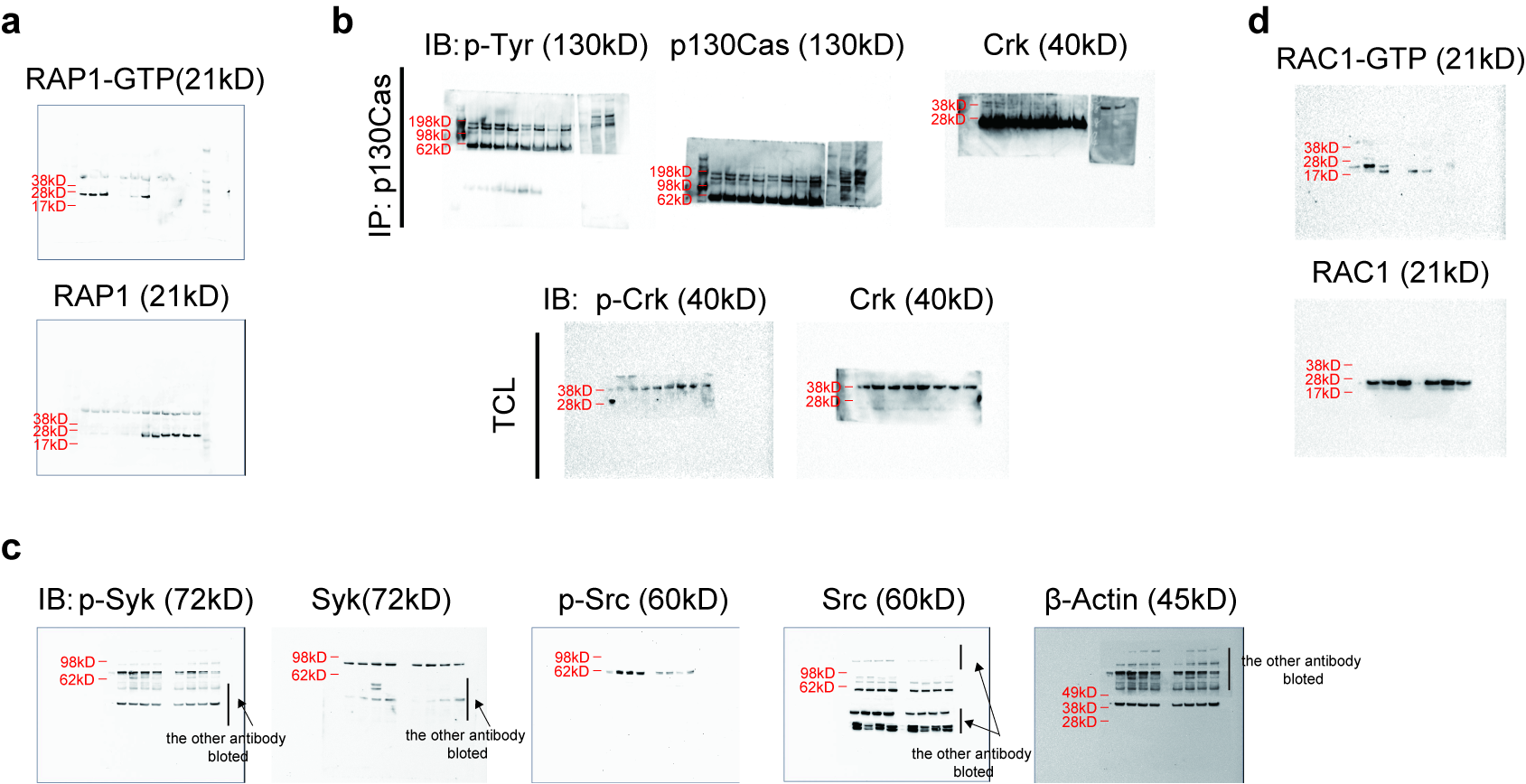

Supplement: Supplementary file 2 — Supplementary Fig. 4 [file 41413_2024_340_MOESM2_ESM.tif]

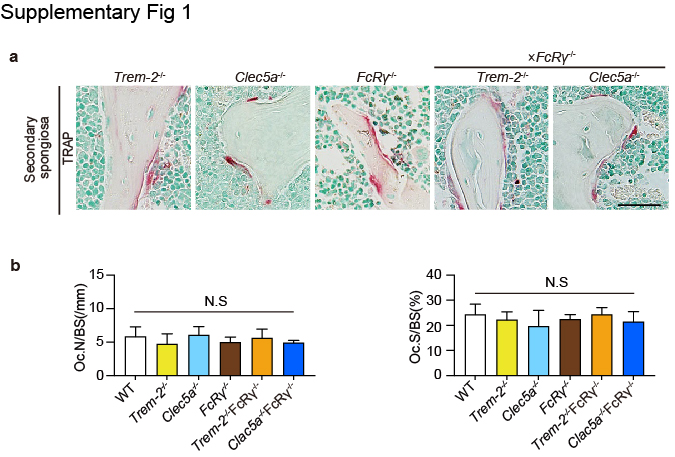

Supplement: Supplementary file 3 — Supplementary Figure 1 [file 41413_2024_340_MOESM3_ESM.jpg]

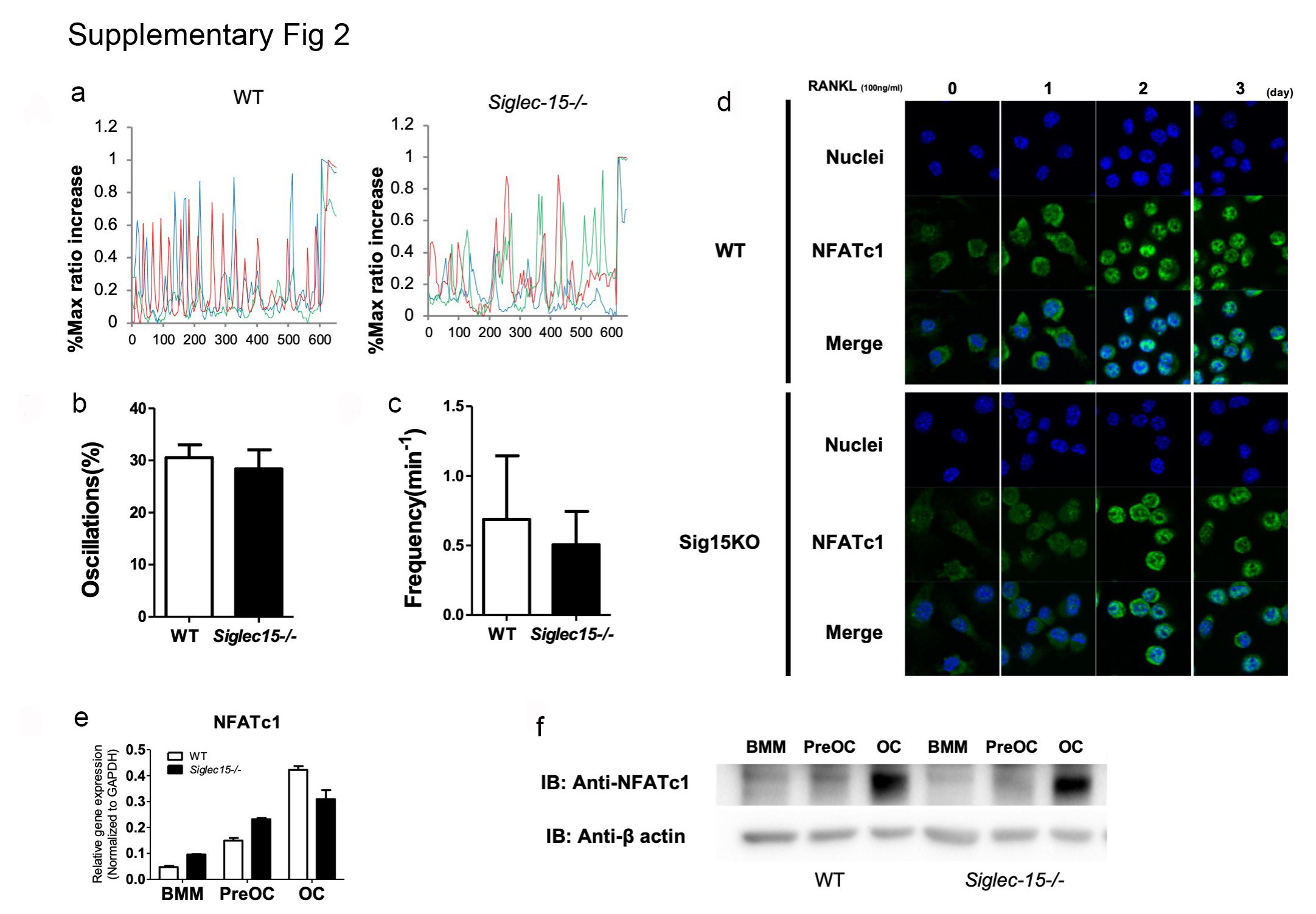

Supplement: Supplementary file 4 — Supplementary Figure 2 [file 41413_2024_340_MOESM4_ESM.jpg]

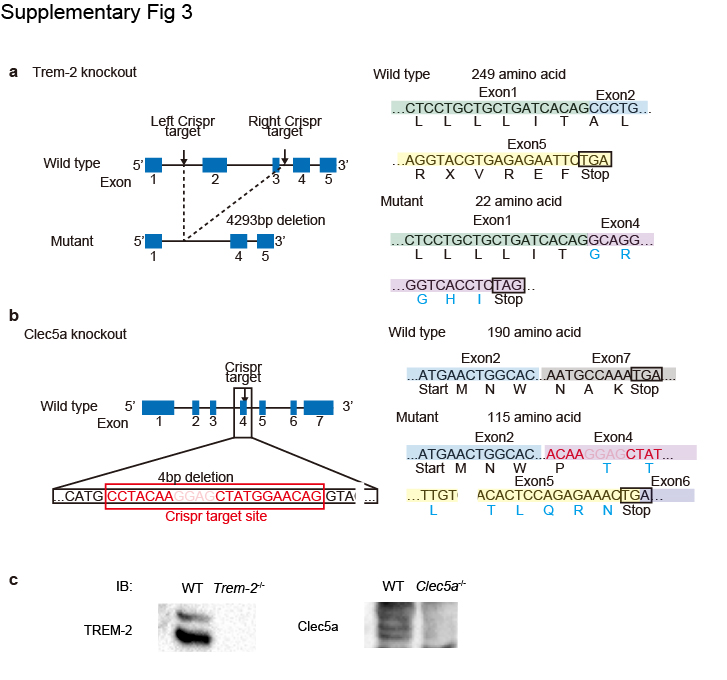

Supplement: Supplementary file 5 — Supplementary Figure 3 [file 41413_2024_340_MOESM5_ESM.jpg]
