# Supplementary material for: Involvement of Siglec-15 in regulating RAP1/RAC signaling in cytoskeletal remodeling in osteoclasts mediated by macrophage colony-stimulating factor
Source: Bone Res. 2024 Jun 7;12:35. doi: 10.1038/s41413-024-00340-w (PMC11161467; doi:10.1038/s41413-024-00340-w)
Supplement: Supplementary file 6 — Supplementary Table [file 41413_2024_340_MOESM6_ESM.pdf]

Supplement Table 1. Sequence of primers used in PCR genotyping or sequence analysis.

Gene	Forward primers (5'-3')	Reverse primers (5'-3')
Siglec-15 Wild type	CTGGTTCCTCAAACTTATCAAACAT	TATGCCTGCTTTTTCTCTGTGAACT
Siglec-15 Neomycin cassette	CGTGCAATCCATCTTGTTCAAT	
FcRγ wild type	CCTACTCTACTGTCGACTCAAG	GGCTGGCTATAGCTGCCTTT
FcRγ Neomycin cassette	CTCGTGCTTTACGGTATCGCC	
Trem-2 wild type	GAGGCTGGAGTCCTGGTACA	GCAGCCTCTTCTCCTACCTG
Trem-2 knockout-	GAGGCTGGAGTCCTGGTACA	TGCACGAAACACATTCCATT
Clec5a Exon4	CAGCAGCCCACAGCCTAATGACTGCTGTGTTCAC	GCATCCTAATGCTTTACCAGTTTCTCTGGAGTG
