# Supplementary material for: Involvement of Siglec-15 in regulating RAP1/RAC signaling in cytoskeletal remodeling in osteoclasts mediated by macrophage colony-stimulating factor
Source: Bone Res. 2024 Jun 7;12:35. doi: 10.1038/s41413-024-00340-w (PMC11161467; doi:10.1038/s41413-024-00340-w)
Supplement: Supplementary file 7 — Supplementary Figure legends [file 41413_2024_340_MOESM7_ESM.docx]

**Supplementary Figure legends**

**Supplementary Fig. 1** Osteoclasts appear morphologically normal in mice lacking *Trem-2* or *Clec5a* and the number of osteoclasts is comparable among mice lacking *Trem-2* or *Clec5a* and WT mice. (a) Micrographs of the secondary spongiosa of the distal femur stained with tartrate-resistant acid phosphatase (TRAP) for osteoclasts. Scale bar = 50 μm. (b) Bone histomorphometry data of osteoclast number and osteoclast surface/bone surface (N.Oc/BS and Oc.S/BS) at the secondary spongiosa of the distal femur (n = 5 per group).

**Supplementary Fig. 2** Siglec-15 has a slight impact on calcium signaling and induction of NFATc1. (a) Calcium signaling in WT BMMs and Siglec15 null BMMs stimulated with M-CSF and RANKL for 3 d. Lines show traces of [Ca^2+^] change in single cells and each color indicates a different cell in the same field of view. Calcium oscillation was observed in both types of cells. (b, c) Percentage of oscillating cells and average frequency of Ca^2+^ spikes were calculated. (d) Nuclear translocation of NFATc1 in both types of cells were shown. Cells were cultured in the presence of M-CSF and RANKL for the indicated period. Localization of NFATc1 was probed with an anti-NFATc1 antibody, which was visualized with a secondary antibody labeled with Alexa Fluor 488. Nuclei were stained with 4',6-diamidino-2-phenylindole. (e, f) NFATc1 expression in WT and Siglec-15 BMMs examined by RT-PCR and western blotting. BMMs were cultured in the presence of M-CSF and RANKL for 3 d.

**Supplementary Fig. 3** Schematic representations of Trem-2 (a) and Clec5a (b) target sites. The genomic region containing exons 2–3 of *Trem-2* was deleted by CRISPR/Cas9 system, leading to flame shift mutation. Four bp nucleotides in exon 4 of *Clec5a* were deleted by CRISPR/Cas9 system, leading to flame shift mutation. (c) Deletion of Trem-2 and Clec5a protein was confirmed by Immunoblotting.

**Supplementary Fig. 4** Molecular weights of each target as analyzed by Western blotting.
